# The LIBRA NeuroLimb: Hybrid Real-Time Control and Mechatronic Design for Affordable Prosthetics in Developing Regions

**DOI:** 10.3390/s24010070

**Published:** 2023-12-22

**Authors:** Alonso A. Cifuentes-Cuadros, Enzo Romero, Sebastian Caballa, Daniela Vega-Centeno, Dante A. Elias

**Affiliations:** Biomechanics and Applied Robotics Research Laboratory, Pontificia Universidad Católica del Perú, Lima 15088, Peru; alonso.cifuentes@pucp.edu.pe (A.A.C.-C.); enzo.romero@pucp.edu.pe (E.R.); scaballa@pucp.edu.pe (S.C.); daniela.vegacenteno@pucp.pe (D.V.-C.)

**Keywords:** brain–computer interface, machine learning, myoelectric control, pattern recognition, sensor fusion, transhumeral prosthesis

## Abstract

Globally, 2.5% of upper limb amputations are transhumeral, and both mechanical and electronic prosthetics are being developed for individuals with this condition. Mechanics often require compensatory movements that can lead to awkward gestures. Electronic types are mainly controlled by superficial electromyography (sEMG). However, in proximal amputations, the residual limb is utilized less frequently in daily activities. Muscle shortening increases with time and results in weakened sEMG readings. Therefore, sEMG-controlled models exhibit a low success rate in executing gestures. The LIBRA NeuroLimb prosthesis is introduced to address this problem. It features three active and four passive degrees of freedom (DOF), offers up to 8 h of operation, and employs a hybrid control system that combines sEMG and electroencephalography (EEG) signal classification. The sEMG and EEG classification models achieve up to 99% and 76% accuracy, respectively, enabling precise real-time control. The prosthesis can perform a grip within as little as 0.3 s, exerting up to 21.26 N of pinch force. Training and validation sessions were conducted with two volunteers. Assessed with the “AM-ULA” test, scores of 222 and 144 demonstrated the prosthesis’s potential to improve the user’s ability to perform daily activities. Future work will prioritize enhancing the mechanical strength, increasing active DOF, and refining real-world usability.

## 1. Introduction

According to World Health Organization statistics, four out of five people with upper limb amputation conditions live in developing countries [1]. This represents about 2.5 million people. In these countries, workers face unsafe conditions without labor insurance, driven by high rates of informality (50% in Latin America) and the prevalence of metal mechanic and extractive industries [2]. In Peru, the Ministry of Labor reports an average of 40 hand and arm injuries per month due to formal occupational accidents [3].

Transhumeral amputations, constituting 2.5% of all amputations [4], involve a residual arm that is used less frequently in daily activities, leading to discomfort due to muscular compensations. As usage decreases, the arm and shoulder muscles atrophy, while adipose tissue increases, potentially causing ankylosis [5]. The loss of movement on one side may result in the complete arm experiencing muscular overload, contributing to long-term lumbar pain [6].

Numerous models of transhumeral prostheses have been developed in recent years, to find a solution to this problem. Mechanical, electronic, and hybrid activation prostheses have been created in academic and commercial fields.

Body-powered transhumeral prostheses, like [7], possess passive degrees of freedom (DOF) and are activated through body movements using mechanisms including buttons on the prosthetic palm, wrist, and elbow. Typically offering two to three DOF at most, these models require coordinated movements between the user’s amputated residual arm and their shoulder or the sound limb shoulder for gesture execution [8]. An example of this control model is [9], a TRL-6 prosthesis developed using 3D printing. Timm’s prosthesis features a harness on the user’s back that, when paired with the movement of the amputated arm’s shoulder, enables movement in two DOF: flexion–extension and pronation–supination, and it can hold objects weighing up to 600 g.

Second, there are electronically activated models where all DOF are started by one or more motors embedded inside the prosthesis. Motors can be placed inside the fingers, in the prosthetic palm, or the forearm and elbow [10]. In addition, these models have one or more embedded batteries that guarantee 4 to 10 h of operation [11]. There are three types of control for these prostheses: through reading physiological signals of electromyography (EMG), electroencephalography (EEG), or shared, with buttons placed locally on the forearm and with remote activation interfaces using a cell phone application. Likewise, the control boards are embedded inside the prosthesis of the palm or forearm. Among the models, ref. [12] presented a prosthesis with TRL-9; it has seven DOF: five in the hand, one in the elbow, and one in the wrist; weighs 913 g; and is made of high-strength plastic. The prosthesis has two EMG sensors, and calibration is performed using a cell phone application.

On the other hand, EEG control models are still under development at the research level. Their purpose is to enable mapping these types of readings, to classify them and convert them into gestures [13]. This control is applied when the EMG reading becomes difficult due to the conditions of the user’s amputated section. In this sense, exclusive EEG control improves the accuracy of the discernment of the gesture [14]. Due to the mapping of brain commands that it applies, it is usually necessary to implement deep learning and machine learning models to decode the EEG data of the headband with an arrangement of sensors operating as a part of the brain–computer interface (BCI) [15]. However, although this has advantages compared to EMG reading alone, the training process for control models usually varies from user to user, in time and comfort [16].

Finally, hybrid activation models control the prosthesis by applying an EMG readout for active DOF and mechanical systems for discrete positions in passive DOF [17]. In this way, the user can preconfigure the passive degrees to achieve the desired discrete position for the activity they want to perform and then rely on the EMG readings to select the type of gesture and its activation. This control is often highly versatile, as it does not overload the muscles around the amputated section and allows the user to sustain a gesture for prolonged periods [18].

Among the hybrid activation models, the work presented by Huang [19] and Ruhunage [20] stands out. Huang proposed the development of a prosthesis with TRL-6 and six DOF (2 in the elbow: pronation/supination and flexion/extension, 2 in the fingers, and 2 in the wrist). The prosthesis weighs 3450 g and can execute up to 12 gestures. In turn, Ruhunage presented a hybrid EMG–EEG control, proposed the development of a prosthesis with TRL-5; seven DOF (6 in the hand and 1 in the wrist, elbow in fixed position); a hand that weights 432 g; hardware pieces made of Polylactic acid (PLA) filaments and aluminum components; and which can execute up to four types of grip.

Regarding the commercial prosthetic models available, the Ottobock [21,22], Fillauer [23], and Segway-Darpa [24] prostheses stand out due to their cost, ranging from 20 K to 100 K USD, which include arm, forearm, and prosthetic hand prostheses. These models can be activated either through sEMG (surface electromyography) or buttons, have a weight ranging from 500 to 900 g (excluding the hand), and offer universal compatibility with various commercial hand models. Customization options are available for purchasers based on their residual limb preferences. Please refer to Table 1 for a side-by-side comparison.

However, despite the technological advances mentioned above, there are two main problems: technical and accessibility. From a technical standpoint, transhumeral mechanical prostheses often require additional compensatory movements from other body parts to achieve the grasping effect, alongside dynamic articulation with the wrist and elbow. These movements increase the level of awkwardness in attaining the final grasping effect [25]. In contrast, electronic models with a more extensive market representation utilize sEMG reading to control the prosthesis (Table 1), which proves efficient for distal amputations (near the elbow) rather than middle and proximal (near the shoulder), as the present muscle shortening [5] results in relatively weaker electromyographic readings. This weak signal fails to reach the high threshold values capable of distinguishing intentional muscle flexions from reflex flexions after undergoing filtering and rectification. In this context, false positives result in increased prosthesis activations. Hence, it is crucial to contemplate adopting a control model that complements the sEMG readings. A complementary control model for the sEMG reading is needed in this scenario.

On the accessibility front, despite all the technological developments mentioned above, these assistive technologies are produced elsewhere. Moreover, the few models produced in developing countries are still in the prototype production phase [26]. In this sense, almost all the local prosthetic offers are imported. In Peru, acquiring a mechanically activated transhumeral prosthesis can cost USD 9000, and prostheses with hybrid control (mechanical and EMG sensors) can cost USD 12,000. These costs are inaccessible in countries like Peru, where the minimum annual salary in the formal market is around USD 4600 [27]. The cost of these prostheses needs to consider the socket customization, the fitting of size and weight, and the functional purpose the user seeks to give to their prosthesis.

Despite the availability of formal insurance policies for limb-loss accidents, a civil process is often required to prove non-negligence and attribute the event to a company accident. Even if covered, insurance typically applies to generic hook-type prostheses for specific amputation levels. With each amputation being unique, factors like evolution time, muscle condition, hypersensitivity, and ankylosis must be considered [28]. Customized models entail pre-prosthetic and post-prosthetic phases, leading to increased development costs.

In summary, regarding the development and accessibility of transhumeral prostheses, the current solutions only partially solve the problem. Due to the level of amputation, they are products that must have a high degree of customization, and to ensure a deep belonging relationship between the user and their prosthesis, high success rates are mandatory. However, all these requirements are currently covered only by the most expensive prostheses, which are highly inaccessible to people with disabilities in developing countries. Therefore, it is necessary to create customizable transhumeral prosthesis models with control methods that—in conjunction with sEMG readings, EEG, and passive discrete DOF placements—represent an accessible and dexterous technology for developing regions.

This work proposes a transhumeral prosthesis with a hybrid control system based on physiological signals. The TRL-3 prototype achieved is a proof of concept addressing the need for accessible assistive technologies in developing regions. A parametric design and digital manufacturing techniques, including 3D scanning and printing, make the prosthesis adaptable to various body sizes and levels of transhumeral amputation. It can be customized based on the user’s weight, to avoid spinal compensations. The model features three active DOF in the prosthetic palm for pinch, lateral, and cylindrical grips, and three passive DOF for the wrist and one for the elbow.

sEMG readings from the residual muscles in the amputated area control the selection and activation of the prosthetic hand’s gestures. In contrast, EEG readings switch the sEMG classification algorithm ON and OFF, ensuring that only intentional movements are executed. This system utilizes concurrent and parallel programming, digital signal processing, machine learning, and Internet of things (IoT) integration for enhanced functionality and control. Training sessions in sEMG and EEG signal management are essential to optimize the user interface with the prosthesis, enabling users to adeptly navigate between mental commands and sEMG readings for effective prosthetic control.

This manuscript is organized as follows: Chapter II outlines the mechatronic design methodology, including the mechanical, electronic, and control subsystems, as well as the hybrid classification model. It also details the protocol for assessing the prosthesis’s functionality. In Chapter III, the results are presented, covering the mechatronic technical performance, the hybrid classification model’s performance, and the experimental validation results with users. Chapter IV interprets these findings, discussing their implications, study limitations, and potential avenues for future research. Finally, Chapter V concludes the study by summarizing key findings and contributions.

## 2. Materials and Methods

The details of the transhumeral prosthesis are divided into four sections. The first section is “Mechatronic Prosthetic Design”, where the components and mechanisms used for the assembly and operation of the prosthesis are described. Next, in the second section, the “System Architecture” is explained, which includes the electrical and control systems. The third section presents the hybrid classification model, starting with the “Data Acquisition Protocol”. Subsequently, the classification models with information from the sEMG and EEG sensors are described, followed by an explanation of the performance evaluation. Finally, the “Prosthesis Functional Assessment” section describes the methodologies used to assess the prosthesis functionality and the user’s ability to perform daily activities. This section describes the experimental protocol participants, configurations, outcome measures, and analysis methods.

### 2.1. Mechatronic Prosthetic Design

In this section, the detailed design of the seven DOF prosthesis is presented. The range of motion encompasses finger flexion/extension, index finger flexion/extension, thumb flexion/extension, thumb abduction/adduction, wrist rotation, wrist flexion/extension or adduction/abduction, and elbow flexion/extension. The prosthesis is structured into three strategically defined modules: hand, forearm, and arm. Three-dimensional printing technology fused deposition modeling (FDM) was used to create the internal and external components. Within the prosthesis, four key components are integrated: the actuation system, the sensing system, the control board, and the power source. The subsequent sections will elucidate the mechanisms and components employed within each module.

#### 2.1.1. Design Objectives and Requirements

The prosthesis must facilitate object grasping through positional gestures to achieve cylindrical or pinch-type grips on thin objects [29,30]. Additionally, for convenience, it should feature an articulated wrist that allows inclined hand positioning for grips that do not require the user to assume an uncomfortable posture. Similarly, the elbow should complement hand positioning for various grips. Furthermore, the user needs to be able to don the prosthesis easily, preferably without requiring assistance from another person.

In total, the complete prosthesis should not exceed 4.5 kg, to avoid imposing excessive weight on the user’s body, and it should have between six and seven DOF [31,32,33]. The plan is to primarily incorporate motor-activated mechanisms in the hand, as this is the primary component for grasping. The remaining mechanisms, such as the wrist and elbow, could be actuated by passive mechanisms. The requirement for durable yet lightweight materials and the need for custom design necessitates working with rapid prototyping technologies like 3D printing. This approach enables the development of a cost-effective and versatile product. Table 2 presents additional design requirements considered in this research.

#### 2.1.2. Enabling Technologies and Design Decisions

The detailed design was based on information gathered about the user’s finger, hand, and arm measurements. In addition, 3D digitization of the amputated arm and the reference arm was carried out using a Revo Point POP 2 scanner. Subsequently, a 3D model was created using the computer aided design (CAD) software Autodesk Fusion 360 (version 16.4.0.2062) which enables processing digitized models of residual limbs. Additionally, Autodesk Inventor Professional was employed to make a parametric and editable design of the complete prosthesis, including the control electronics board.

The primary manufacturing technology for components in this prosthesis was FDM 3D printing. Prototypes of the main parts and external covers were created using PLA material, while the internal mechanisms were crafted in acrylonitrile butadiene styrene (ABS) material. Based on specific functions, load-bearing components were manufactured with 40% infill and shells and covered with 15% infill. In addition, PLA molds were used to create fingertips made of platinum silicone RTV-1510 from SILIKAMOLDS; these enhanced the coefficient of friction.

Additional securing elements, such as Velcro straps, were custom-made by taking extra measurements of the participants’ residual limbs and chest. Traditional sewing methods were used to create a harness with elastic straps, nylon webbing, and thick textile neoprene.

Finally, the control board was designed using EasyEDA. After manufacturing the board, the firmware was loaded, and the coding was performed using Python, C, and C++ programming languages in the Visual Studio Code and PlatformIO programming environments. Subsequently, the control parameters of the prosthesis were fine-tuned (actuator movement speed, extreme limits of motion, and control logic). These programming tools were also used to develop and edit the integrated gesture classification system.

#### 2.1.3. Hand Module

**Figure 1:**
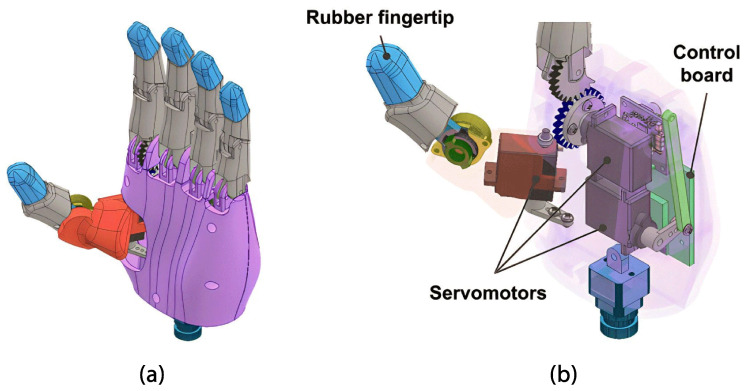
Internal and external views of the prosthesis: (**a**) hand prosthesis assembled, (**b**) internal components.

**Figure 2:**
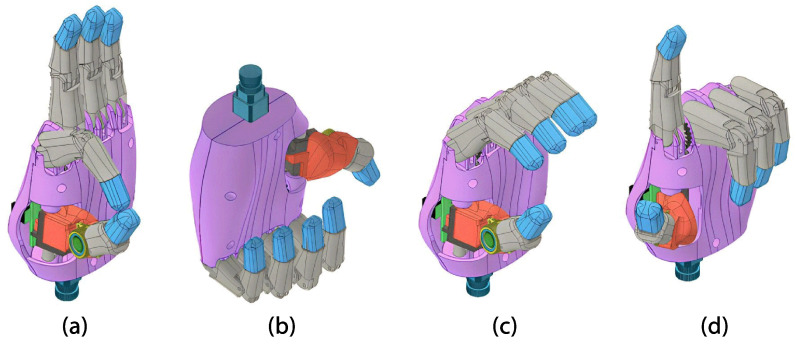
Grip types and gestures: (**a**) cylindrical, (**b**) hook, (**c**) pinch, (**d**) finger-pointing/pressing.

The hand module consists of a palm that securely holds four fingers at the top, with the thumb positioned at the base of the palm. In this case, the left hand will match the user’s amputation. On the inside, servo motors and the control board are placed as shown in Figure 1. Furthermore, actuators guarantee cylindrical, hook, and pinch grasping. In addition, the prosthesis can perform finger pointing and pressing (see Figure 2).

##### Fingers

**Figure 3:**
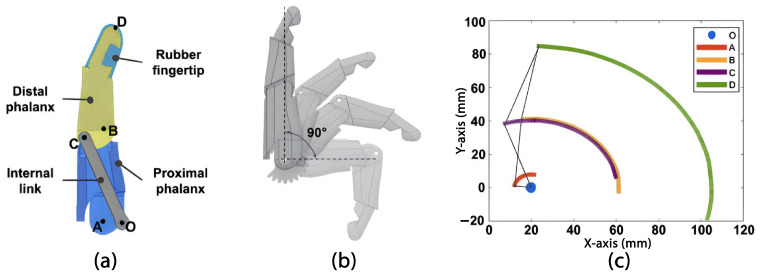
Finger mechanism: (**a**) previous underactuated mechanism with joints “A”, “B”, “C”, and the top of the fingertip “D” (**b**) finger transition, (**c**) points “A”, “B”, “C”, and “D” trajectory in a sagittal plane.

The design of the fingers in our latest model was based on our previous work in body-powered prostheses [34] In this approach, we use the total length and breadth of each finger from either hand of the user as a reference. Based on these measurements, CAD assembly files for the fingers are automatically generated. The design considers a medial-distal phalanx and a proximal phalanx. Additionally, the internal 4-bar linkage mechanism (see Figure 3a), designed following the Grashoff equations, was described in a previous study on anthropomorphic hand prostheses [35]. Taking the index finger as a reference, it is possible to analyze the motion trajectory of each of the marked points placed on rotational joints (“A”, “B”, and “C”) and the top of the fingertip (“D”) concerning a relative fixed point “O”. The finger’s displacement from the position of full extension to the position of full flexion results in a finger angular displacement of 90° (see Figure 3b). Furthermore, the graph shown in Figure 3c illustrates the position of each point throughout the entire trajectory, in millimeters, in the sagittal plane, where it is evident that at maximum flexion, the fingertip (point “D”), is located below the fixed pivot point “O”.

Research into activation mechanisms powered by a single actuator for these fingers revealed the necessity for a complex mechanism and the limitations regarding achieving adaptive grips and incorporating more than two grip types in a prosthesis [36,37]. The previous prototype of the LIBRA NeuroLimb’s prosthetic hand was underactuated, driven by a single DC motor that flexed or extended the finger digits II, III, IV, and V [35]. The primary challenge in implementing this mechanism was the significant stresses imposed on the internal plastic components, reducing durability. This experience informed the transition from DC to servo motors and to more direct transmission mechanisms.

**Figure 4:**
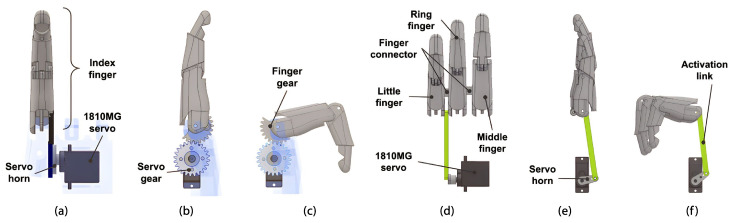
Index finger and finger group gear transmission: (**a**) servo and index finger connection, (**b**) index finger maximum extension, (**c**) index finger maximum flexion, (**d**) servo and fingers connection, (**e**) fingers maximum extension, (**f**) fingers maximum flexion.

Because the index finger exerts the most effort during object grasping and the prosthesis must be capable of performing pinch-type grips, it was decided that it should be independently actuated using a direct transmission powered by a Power HD 1810MG (Santa Barbara, CA, USA) 3.9 kgf-cm servo motor (see Table 3) through a gear transmission composed of a servo horn and a servo gear jointed with four 2.5 mm screws, with a reduction ratio of 1:1 (see Figure 4). The finger mechanism uses internal 2 mm stainless steel shafts to articulate the 4-bar linkage to perform a 90° range of movement.

The middle finger, ring finger, and little finger are interconnected and move synchronously, due to the fingers being linked by the finger connector. Figure 4 illustrates that the activation link for this group of fingers is applied on the underside of the little finger, and this link transmits the force exerted by a second Power HD 1810MG servo motor, which supplies 7.28 kgf-cm to the little finger.The torque applied to the fingers is calculated considering 2 mm stainless steel shafts for the rotational joints, the 3.9 kgf-cm of the servomotor transmitted by the 12 mm servo horn, and the 59 mm activation link. Table 3 shows a servo motor specification comparison.

##### Thumb

**Figure 5:**
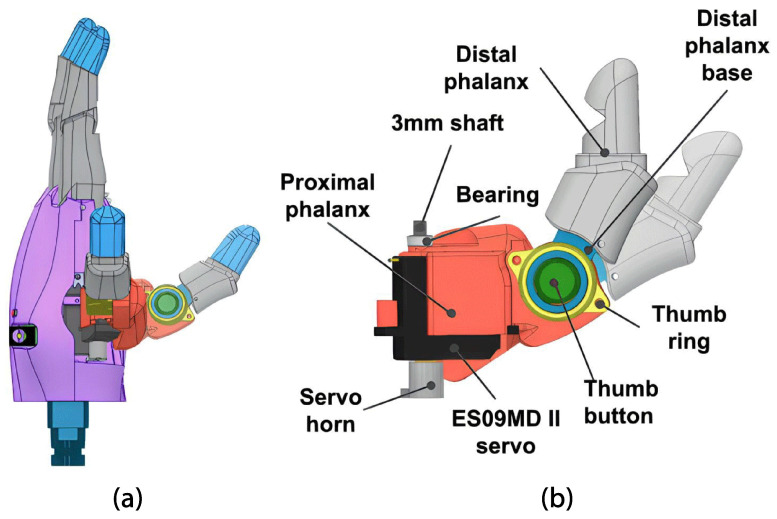
Thumb mobility and parts: (**a**) thumb flexion/extension, (**b**) thumb abduction/adduction.

As a requirement, the thumb needs to be capable of performing cylindrical and hook grasps. For this reason, the thumb is fixed to the palm using a rotary joint connected to an EMAX ES09MD II (Dubai, United Arab Emirates) 2.6 kgf-cm servo motor (see Table 3), which facilitates abduction and adduction movements. This actuator is attached to the thumb’s proximal phalanx. At the top, it is connected to a 3 mm shaft and a 3 × 6 × 2.5 mm bearing, and at the bottom, it is attached to a servo horn using two 2.5 mm screws (see Figure 5). Furthermore, the open-source HACKberry Exiii Inc. (Tokyo, Japan) thumb mechanism for abduction/adduction was incorporated to expand or contract the thumb distal phalanx to 30°, expanding the gripping capacity for objects of varying dimensions during the cylindrical grip.

##### Palm

The hand, designed using the same technique as the fingers, requires input for the length and breadth of the palm. The fingers and thumb are securely attached to the palm, with servo motors in cavities fastened using 2.5 mm screws. The control board is securely attached using three strategically placed 2.5 mm screws, for external access. A wrist connector at the palm’s base establishes the connection between the hand and the wrist.

**Figure 6:**
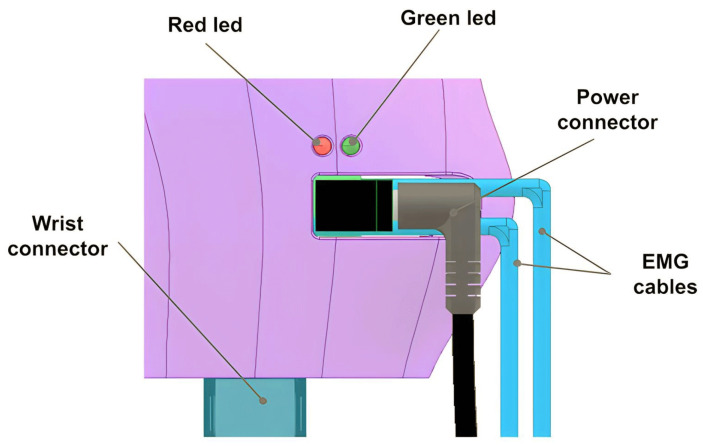
LED indicators, sEMG cables, and power connector.

The board includes two LED indicators. The illuminated red LED indicates that the sEMG sensor readings in the prosthetic socket will not influence the prosthesis operation. Conversely, when the green LED is lit, the sEMG readings will be utilized to control the movement of the prosthesis fingers. Finally, the power connector, positioned externally, establishes the hand–wrist connection. The sEMG sensor wiring also enters through the palm’s sides (see Figure 6).

#### 2.1.4. Forearm Module

**Figure 7:**
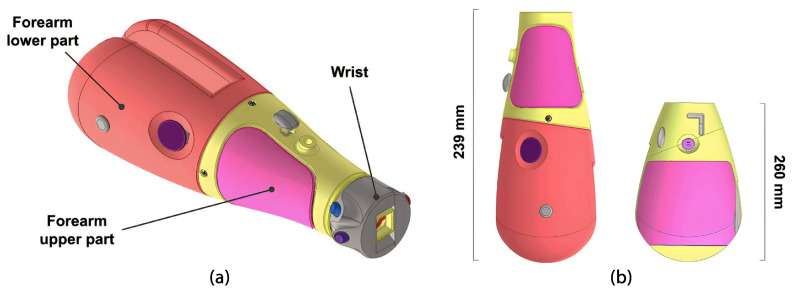
Forearm: (**a**) components, (**b**) designs.

This module enables dynamic positioning of the palm and elbow flexion. Activation is primarily passive, and the module also houses and safeguards the power source system internally. The buttons have been strategically positioned to allow easy access for the user with their other hand. Figure 7a shows the main components of the forearm module. In this study, two forearm models were designed, each with varying lengths, to accommodate the individual measurements of the original user’s body. Figure 7b shows both designs and their different dimensions.

##### Wrist

**Figure 8:**
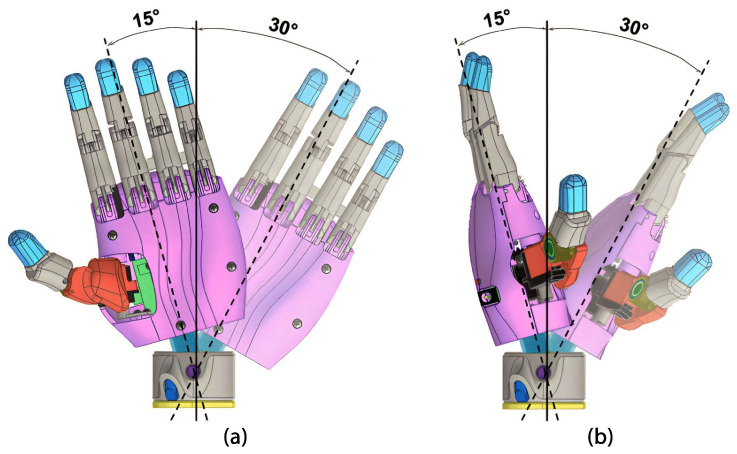
Wrist connected to the hand: (**a**) adduction/abduction trajectory, (**b**) flexion/extension trajectory considering 90-degree wrist connection alternation.

**Figure 9:**
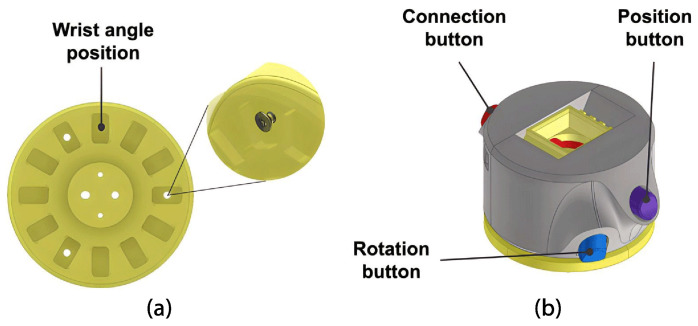
Wrist principal parts: (**a**) wrist base and rotation positions, (**b**) wrist and the principal buttons.

An open-source HACKberry Exiii Inc wrist model was implemented for the wrist mechanism. This wrist features three buttons that can be manipulated. The connection button, when pressed, attaches or detaches the hand from the wrist; the position button allows adjustment of the angle for adduction/abduction positioning from −15° to 30° in four different stages (see Figure 8a), and when the hand is connected with a 90-degree rotation it controls the wrist flexion/extension angle from −15° to 30° (see Figure 8b). The rotation button facilitates wrist rotation and fixes the rotation angle in twelve different positions (see Figure 9). The upper part is attached to the wrist using three screws.

##### Forearm

**Figure 10:**
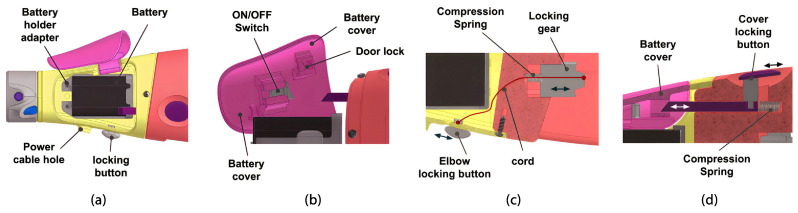
Forearm upper detail: (**a**) internal battery and its supports, (**b**) additional components from the battery cover, (**c**) elbow locking system, (**d**) battery cover locking system.

The forearm is parametrically designed to match the user’s forearm length and width and is divided into two sections: the upper part and the lower part. The main cover provides protection and access to the rechargeable battery inside the battery holder adapter. This battery is connected to an on/off switch button on the cover; see Figure 10. The battery and switch circuit routes power through a cable, covered with heat-shrink tubing, extending from the upper forearm housing and terminating in a jack-type connector, which powers the control board. The door lock slot demonstrates in high relief how the silhouette of the switch looks when it’s in the “on” position, to facilitate the understanding of the end user.

**Figure 11:**
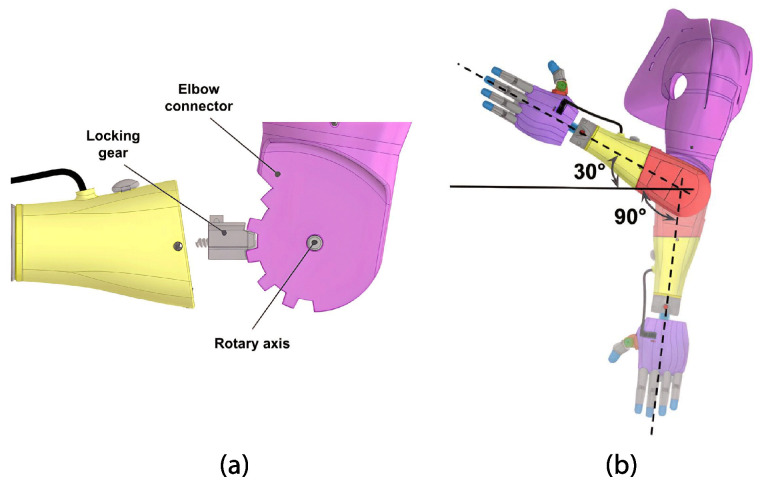
Elbow and arm: (**a**) elbow locking system, (**b**) arm tilt positions.

The lower part includes a retractable mechanism that secures the upper part’s cover in the closed position (see Figure 10). Additionally, in the center of this component, there is a movable piece that functions as a locking gear and is actuated using a cord connected to the elbow locking button located on the surface of the upper part of the forearm. Both mechanisms include a stainless steel compression spring designed with 0.5 mm wire diameter, 6 mm out diameter, and 8 mm length. Finally, the forearm is fixed to the arm module via a rotary axis, creating the elbow of the prosthesis (see Figure 11).

#### 2.1.5. Arm Module

The third module is crucial for users, as it not only supports the weight of the other modules and carried objects, but also requires a comfortable prosthetic socket. To achieve this, personalized measurements of the residual limb, including lengths and diameters, are taken, and a 3D scan enables the design of a customized socket. The arm module has two main components: the elbow connector and the body attachment.

The elbow connector is attached to the lower part of the forearm, as shown previously. Figure 11 details the connection between the locking gear and elbow connector. Activation of the forearm locking button allows switching between five tilt positions for the elbow from −30° to 90°, as shown in Figure 11b. This mechanism was adapted from an open-source design from Po Paraguay.

**Figure 12:**
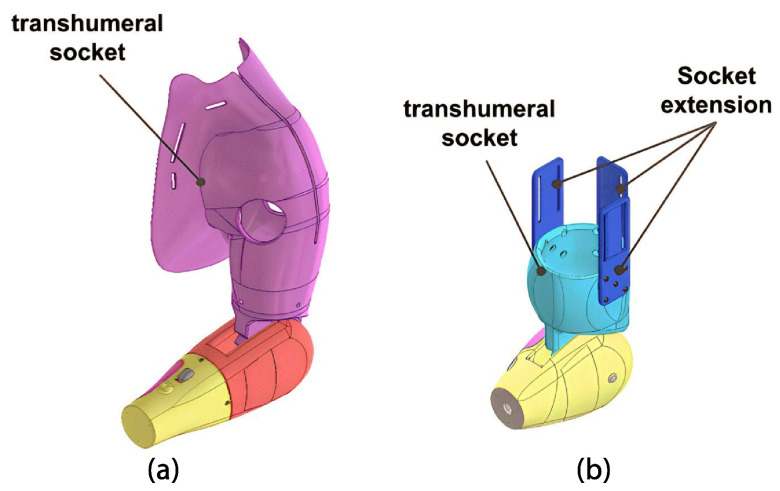
Body attachments designed: (**a**) transhumeral socket, (**b**) short transhumeral socket.

The body attachment is designed to fit the user’s residual limb, with two types developed in this research. The first, for a transhumeral amputation, includes a socket accommodating chest measurements for improved prosthesis stability (Figure 12a). A harness with Velcro straps is used to prevent slippage. For a short transhumeral amputation, the second features petal-like pieces for securing Velcro around the residual limb (Figure 12b). Both designs incorporate internal cavities for sEMG sensors.

### 2.2. System Architecture

#### 2.2.1. Electrical System

**Figure 13:**
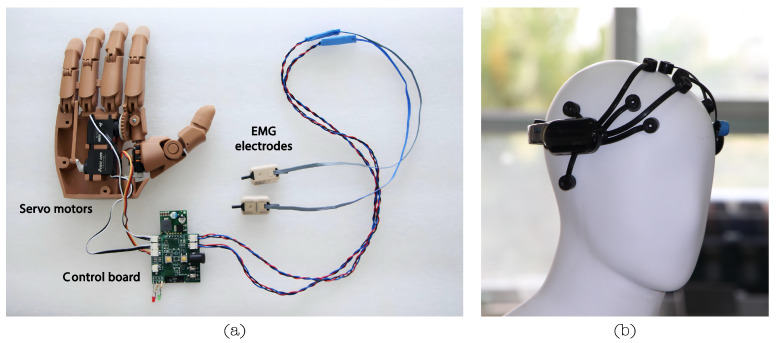
Main electrical components: (**a**) internal palm components, (**b**) external BCI device.

**Figure 14:**
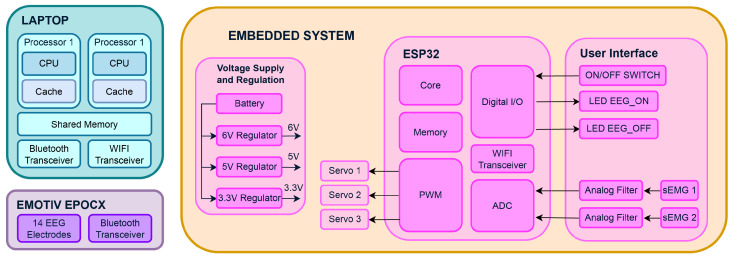
System architecture. Abbreviations: analog-to-digital converter (ADC), pulse width modulation (PWM), electroencephalography (EEG), and surface electromyography (sEMG).

The LIBRA NeuroLimb prosthesis is a transhumeral prosthesis with electromyographic and electroencephalographic signal control capabilities. Although it possesses an embedded system, it has a modular design. Figure 13a shows the control board connected to the servo motors and the sEMG electrodes. Therefore, external devices such as an EMOTIV EPOC X EEG (see Figure 13b) and a computer or laptop are required to achieve maximum functionality. Figure 14 shows an overall system architecture schematic, including the EEG modules and an external laptop.

An sEMG and EEG-based system was chosen for transhumeral amputees, due to the impracticality of retrieving signals from forearm muscles. To enhance the precision and data dimensionality, signals from adjacent muscles (biceps, triceps, shoulders, chest, and back) are utilized for classification [38,39]. While relying solely on bicep and tricep signals might require specific sequences for gesture selection, a multi-sensor system compensates for accuracy and prediction time limitations. Sultana’s analysis [40] suggests an improved accuracy with more than 2 electrodes but a decline with 12 or more. A multi-sensor system addresses these challenges and facilitates the integration of advanced features like a brain–computer interface and using sEMG sensors for gesture selection, enhancing the precision and usability [16,41,42,43].

Starting with the description of the embedded system, the prosthesis has a 7.2 V 2100 mAh battery (model DMW-BLF19), which powers the main board that has a power regulation system consisting of 3 step-down regulators with output voltages of 3.3, 5, and 6 V to power the main microcontroller, integrated circuits, and servomotors, respectively. The main board has the function of acquiring the sEMG signals from the user’s amputated arm, controlling the servomotors corresponding to the movement of the fingers, and sending and receiving the sEMG and control signals via WIFI to the laptop. The main microcontroller is an ESP32-WROOM-32D-N16, a wireless microcontroller module for IoT applications. It features a dual-core processor running up to 240 MHz, built-in Wi-Fi and Bluetooth connectivity, 32 MB of flash memory, 448 KB of ROM, 520 KB of SRAM, and various peripherals. It operates at a voltage range of 2.2 V to 3.6 V, has low-power-consumption capabilities, and includes security features like secure boot and cryptographic accelerators. Meanwhile, the laptop used in this project had the following characteristics: 11th Gen Intel(R) CORE(TM) i7-1165g7 @ 2.80 GHz 1.69 GHz, 16 GB RAM, and 64-bit processor, with an NVIDIA GeForce MX450 GPU (Santa Clara, CA, USA).

The sEMG signals are captured using two non-invasive Ottobock 13E200-60 electrodes (Duderstadt, Germany). They weigh 4.5 g and have 27 × 18 × 9.5 mm dimensions. These electrodes operate between 4.8 V and 7.2 V, with a frequency bandwidth of 90 to 450 Hz, and function in temperatures from −15 to +60 degrees Celsius. One electrode measures the bicep’s electrical activity, and the second measures the tricep’s activity, with a variable sensor placement based on the user’s limb anatomy and prosthesis design. It is essential to highlight that the sensors are positioned directly on the user’s skin surface in the bicep and tricep areas. As the sensors give a 0 to 5 V signal, a low-noise operational amplifier adjusts the output to 0 to 3.3 V to protect the microcontroller. Ottobock sensors have internal preamplification, rectification, and smoothing, requiring preprocessing to recover frequency content. sEMG signals are acquired at 12-bit resolution and a 100 Hz sampling rate.

On the other hand, EEG signal acquisition was performed with an EMOTIV EPOC X. This device is a low-cost headset with 14 saline-based electrodes (AF3, F7, F3, FC5, T7, P7, O1, O2, P8, T8, FC6, F4, F8, and AF4) plus two reference electrodes that are widely used for academic and research purposes. Like other devices with the same electrode distribution, it does not have electrodes in the motor cortex area. In addition, the EMOTIV BCI-OSC (version 3.6.5.262) (San Francisco, CA, USA) interface was used to train mental commands. This software allows the user to train mental commands and send real-time information about those mental commands, such as class and accuracy, to an external device via OSC (open sound control) packets.

The prosthesis achieves its motion through the actuation of three servomotors: a Power HD 1810 for the index finger; another Power HD 1810 for the middle, ring, and little fingers; and a EMAX ES08AII for the thumb. These servomotors are controlled using the PWM channels of the ESP32. Detailed technical specifications for the EMAX ES08AII and the Power HD 1810 can be found in Table 3.

Finally, an ESP32 internal WIFI data transceiver is used for communication between the microcontroller and the laptop. This transceiver is used to send messages in the UDP (user datagram protocol) protocol (connectionless), due to the high data transmission speed it allows at a relatively low to medium level of security.

The main microcontroller, powered by the lightweight and configurable FreeRTOS real-time operating system, concurrently manages four essential tasks. FreeRTOS is renowned for its quick task execution, making it suitable for applications requiring rapid responses. It supports various architectures and offers task scheduling, inter-task communication, and efficient memory management. The first task involves acquiring and storing sEMG signals in memory at a fixed rate. Tasks two and three handle communication over a UDP connection—sending predefined values and managing incoming information. This streamlined setup expedites encoding and decoding processes. The fourth task translates received state variables into physical movements, executing selected gestures through servomotors. Further details on these state variables and their role in motion control are provided in Section 2.2.2.

The laptop receives and processes the sEMG and EEG signals to predict the state of the prosthesis (ON/OFF) and the gesture to be performed by the prosthesis in real time. Likewise, the sEMG and EEG classification algorithms are trained with this same device through a graphical user interface. This interface allows the visualization of sEMG signals in real time, to select the parameters of the classification model, which will be discussed in Section 2.3, and consequently to train each classification algorithm.

On the other hand, the training and execution of EEG-based mental commands were performed using the EMOTIV BCI application and the EMOTIV BCI-OSC extension, with which OSC packages were obtained using Python code. It should be noted that all the programs used for the real-time operation of the prosthesis and the training of the sEMG and EEG signal classification models were written exclusively with Python code and freely available libraries.

#### 2.2.2. Control System

The LIBRA NeuroLimb prosthesis employs a multi-process and multi-threaded control architecture to ensure efficient operation and high adaptability. This architecture comprises two primary processes. The first is a Python-based system that leverages multiprocessing capabilities, enhancing its ability to handle diverse tasks. The first process manages communications within this system, seamlessly supporting OSC and UDP communications through dedicated threads. This capability is significant for real-time data transfer and integration with devices like the EMOTIV EPOC X and the ESP32-WROOM-32D module. On the other hand, the second process is responsible for critical functions, including signal processing, AI prediction, and prosthesis control. This process employs thread optimization for tasks such as signal preprocessing, feature extraction, AI prediction, and control. The Python code relies on several open-source libraries that are essential to its core functionality:

- Threading: The ‘threading’ library manages concurrent real-time data handling and communication tasks;
- Multiprocessing: ‘Multiprocessing’ facilitates parallel task execution, which is crucial for coordinating concurrent processes, especially in real-time data acquisition and control;
- Socket: ‘Socket’ enables network communication, ensuring seamless data exchange and integration with external devices;
- NumPy and Pandas: These libraries are essential for multidimensional data operations, manipulation, and processing, forming the foundation of efficient data handling;
- SciPy: ‘SciPy’ manages statistical and signal processing operations, augmenting data analysis and signal manipulation capabilities;
- pythonosc and argparse: These libraries enable communication via OSC and support interactions with external systems and devices;
- Scikit-Learn (sklearn): ‘Scikit-Learn’ empowers the code to efficiently perform classification and data processing tasks, offering a wide range of capabilities, including real-time data handling and classification.

Simultaneously, the ESP32 module, equipped with a dual-core structure, interfaces seamlessly with the primary Python-based system. The first core manages communication tasks, while the second is designated for control functions. This arrangement ensures real-time coordination between the microcontroller and the laptop.

Signal acquisition starts with two sEMG electrodes and conditioning through an analog filter. The ESP32 captures and transmits signals to the laptop over WIFI using UDP. The subsequent preprocessing involves techniques like window-overlapping and normalization. Feature extraction derives time and frequency domain features, preparing data for classification. A support vector machine (SVM) classifier then distinguishes three upper-arm gestures: neutral state, bicep contraction (hand open/close), and tricep contraction (hand gesture change). The preprocessing, feature extraction, feature selection, and gesture classification details will be discussed in Section 2.3. Before classification, the system verifies the prosthesis’s operational state, determined using a mental command from EMOTIV EPOC X relayed via EMOTIV BCI-OSC. The BCI–EEG code operates in latching mode, maintaining the prosthesis state until a new mental instruction is received. The SVM is engaged for gesture prediction only when activated.

After successful ON state verification, the sEMG classification task is enabled. If a new class is predicted, the system communicates the specific hand or grasp command to the ESP32. For safety, the ESP32 checks the prosthesis state and the novelty of the command before directing the servomotors. The servomotors maintain their previous positions without a new command, ensuring user safety and device reliability.

Two state variables govern the servomotor control. The primary state determines the open or closed position of the gesture, while the secondary state specifies the type of grasp, such as cylindrical or pinch. Command initiation originates from the laptop, with the ESP32 executing the desired actions.

### 2.3. Hybrid Classification System

**Figure 15:**
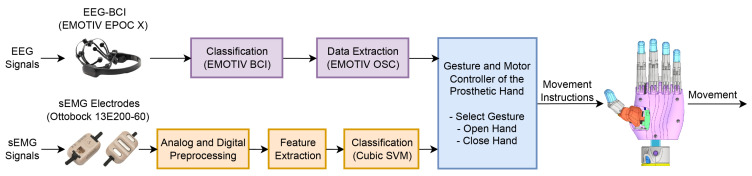
Simplified flow diagram of the hybrid control system in the LIBRA NeuroLimb. Abbreviations: brain–computer interface (BCI), electroencephalography (EEG), surface electromyography (sEMG), and support vector machine (SVM).

The Hybrid Classification System in the LIBRA NeuroLimb prosthesis integrates sEMG and EEG to provide enhanced prosthetic control for transhumeral amputees. This system captures, processes, and classifies the sEMG signals from the user’s biceps and triceps, enabling the selection and execution of prosthetic hand gestures. It facilitates the management of hand functions, including opening, closing, and transitioning between different grips. Concurrently, the EEG-based brain–computer system enables the user to toggle the sEMG classification algorithm on and off, ensuring that the prosthetic arm responds only to deliberate movements, thereby preventing unintended actions and enhancing the overall functionality of the prosthesis. Figure 15 shows a simplified flow diagram of the prosthesis’ hybrid control system, emphasizing sEMG and EEG signal processing and classification for hand gesture control.

This section outlines the sEMG and EEG data acquisition protocol, including the participant description, session setup, and sensor placement. It also provides insights into the hybrid classification model, primarily focusing on the sEMG feature extraction, selection, and classification algorithm design and optimization.

#### 2.3.1. Data Acquisition Protocol

##### Participant Description

The study involved two adult participants: volunteer OB, a 50-year-old man with a distal transhumeral amputation, and volunteer LV, a 36-year-old woman with a proximal transhumeral amputation. Inclusion criteria specified an age range of 18 to 50 years and non-congenital transhumeral amputation resulting from severe trauma, infection, or a medical condition. Exclusion criteria included neurological diseases, motor disabilities, sensory impairments, and cognitive or psychological disorders that could significantly impact the study outcomes. Neurological and clinical evaluations were conducted to verify the participants’ eligibility based on these criteria, following prior research like Li et al. and Kim et al., who developed classification algorithms for prosthesis control using sEMG and EEG signals [44,45]. This study acknowledged variations in participants’ amputation characteristics and leveraged the Biomechanics and Applied Robotics Research Laboratory (LIBRA) network for participant selection, ensuring informed consent.

##### Session Setup and Sensor Placement

The signal acquisition sessions were divided into two sections: the first was to acquire sEMG signals, and the second was for EEG signals. In the first part, the main control board, described in Section 2.2.1, was employed to sample the analog output signals from two Ottobock 13E200 = 60 sensors at a sampling frequency 100 with a 12-bit resolution. A customized sensor placement was conducted in the region with the most significant muscle activity on the amputated arm skin surface of the remaining biceps and triceps muscles, to ensure the highest quality of sEMG signals for subsequent processing and classification. This involved mapping the sEMG activity at various points on the arm to identify areas with the best signal-to-noise ratio, highest power, and optimal signal waveform. This individualized process was applied to each participant, to guarantee the highest signal quality.

In addition to the control board and sensors, a 7.2 V power supply was used as a battery replacement, along with a laptop computer. The laptop was connected via UART to the signal acquisition board to receive, display, and save data from both channels in a CSV file. Simultaneously, data from both channels were displayed in a graphical user interface, which was employed to visualize sEMG signals during training and identify executed commands. This information was later used for training the signal classification algorithm.

The EEG signal acquisition section involved using the EMOTIV EPOC X headwear, which transmitted EEG data via Bluetooth to a laptop. The laptop was used to run the EMOTIV BCI program and the EMOTIV BCI-OSC extension, allowing real-time training data storage. For the placement of the EPOC X, the standard placement guidelines recommended by EMOTIV were followed, without making any specific adjustments for each participant. In addition to all this equipment, cameras were utilized to document the process.

##### Experimental Procedure

Participants were scheduled for signal acquisition sessions for 1 h and 30 min. Trials were executed in controlled environments. The following sequence was directed: equipment preparation, sensor placement, sEMG signal acquisition, and EEG signal acquisition. These stages are described further below. The protocol used in this study was approved by the Committee on Research Ethics for Life Sciences and Technology through dictum 006-2022-CEICVyTech/PUCP. In the equipment preparation stage, it was verified that all the hardware was functional and energized and that the sEMG and EEG electrodes were correctly placed and made proper contact with the participant’s skin.

In the sEMG signal acquisition, participants sat comfortably facing a screen displaying their arm’s sEMG activity. They executed three upper arm muscle contractions—bicep, triceps, and bicep + tricep isometric—corresponding to hand opening, hand closing, and gesture change. Each participant completed ten arm movements in series, with one-minute breaks. Each series included 15 consecutive arm movements: five bicep contractions, five triceps contractions, and five isometric contractions. Participants started from a relaxed arm position, performed the designated contraction for up to 1 s, and returned to the initial position. Arm movements began promptly upon an auditory cue from the overseeing researcher, and the total duration of this section was less than 20 min.

The objective of the EEG data acquisition section was to train two mental commands: the first corresponding to enabling the gesture classification system, and the second related to turning off the gesture classification system. The EMOTIV BCI application was used for this objective, which allows the training of mental commands using the 14 electrodes of the EPOC X. For this purpose, the participants were seated in front of the LIBRA NeuroLimb’s prosthetic hand, with 2 LEDs (green and red) representing the enabled and disabled states, respectively. During the training of the first command, the red LED was initially turned on at maximum intensity, and the green LED was turned off. The user’s objective was to turn on the green LED (turn on the system), and the higher the intensity of the thought or intensity of the command, the more the green LED would turn on, and if they stopped thinking about the command, the red LED would turn on, and the green LED would turn off. The operation of the LEDs was reversed for the second command, in which the objective was to turn off the classifier, so the green LED initially started on.

Regarding the training sequence, there were eight training sets with 1 min breaks between each set. Each training series comprised the training of 5 repetitions of the neutral command, five repeats of the enable command, and five repetitions of the disable command. The neutral command required the participant to be in a state of rest or tranquility. This allowed the algorithm to more accurately identify when the user was not executing one of the other two mental commands. For the repetition training, the sequence was as follows: the participant was informed of the start of the repetition; three beeps were made, signifying the start of data recording; data were recorded for 8 s; and a 3 s wait before starting the next repetition. It was crucial that participants were directed to perform each movement or mental command collaboratively in both the sEMG and EEG sections. In this regard, they performed at least one series of test training for each type of sensor. This ensured that the rest of the acquisition series would be of the best quality.

#### 2.3.2. Hybrid Classification Model

This section examines the methodologies employed for the sEMG and EEG gesture classifications. However, the primary focus remains on the sEMG gesture classification algorithm. Before sEMG signal classification, sEMG signals were preprocessed and segmented, focusing on real-time implementation. Initially, eighteen features were extracted from the sEMG signals, but feature selection optimization refined this set to ten. An SVM classifier was chosen based on its demonstrated precision and efficacy in previous applications [46,47]. This approach included the systematic training, evaluation, optimization, and selection of the best classification algorithm and its corresponding parameters and hyperparameters. Considering individual participant variability, this methodology balanced the accuracy and efficiency in gesture classification.

Regarding EEG signal classification, EMOTIV BCI and its OSC plugin handled training, real-time execution, and accuracy tracking, without extra processing. The control code triggered classifier activation or deactivation based on the command type and accuracy. Activation occurred when the command and accuracy exceeded 75%, given this differed from the preceding valid command. Deactivation happened in similar circumstances. The system’s status was stored in shared memory. To assess the EEG classification accuracy, the sampled command accuracy and EMOTIV BCI’s average accuracy were analyzed after each cycle.

In the upcoming sections, a comprehensive understanding of the employed methodology will be provided, and more details about the preprocessing, feature extraction, feature selection, and sEMG gesture classification stages will be presented.

##### Preprocessing

The Ottobock 13E200-60 sEMG sensors’ signals underwent analog and digital preprocessing. Analog preprocessing involved signal conditioning to match the analog-to-digital converter’s safe operating level using a low-noise OPAMP LM324N from Texas Instruments (Dallas, TX, USA).Subsequently, the signals were digitized at 100 Hz with a 12-bit resolution.

As the sensors internally incorporate preprocessing filters, including a bandpass filter (90 Hz to 450 Hz), rectification, and signal smoothing, the sensor’s output signal is not a raw sEMG signal. Still, it was transformed and distorted in time and frequency. This transformation had to be considered to avoid over-distorting the signal during further preprocessing and feature extraction, especially when conducting frequency domain analysis.

The digital preprocessing and feature extraction process were consistent across all participants.

Previous studies showed that when working with sEMG signals corresponding to active muscle activation, using a standard window of 300 ms is sufficient to obtain a correct characterization of the signal, since shorter window lengths, such as 125 ms, tend to cause a high variance in the signals and increase the error of the classification models [48]. In addition, regarding window overlapping, studies tend to use, on average, an overlapping of 50% of the window length [49,50,51]. Considering a 300 ms window, the overlapping duration would be 150 ms. Considering these factors and due to the average wavelength obtained with the sensors used in this study (>1 s), and because the objective was the design of a real-time system with a fast update of the prosthesis states, it was decided to use windows of 2 s or 200 samples and an overlapping of 25 samples, corresponding to 250 ms, considering a sampling frequency of 100 Hz. These values ensured that, in the event of a muscle contraction, it was contained within the length of the window. For this purpose, a synchronization signal was recorded manually during the acquisition session. In addition, the overlapping length ensured that the time in which a prediction could be made was reduced, as with a model with shorter window lengths. A total of 800 samples per participant were gathered from this procedure. Of these, 75% were designated for training the model, and the remaining 25% were used for validation. A strategy to address the class imbalance was implemented, involving using an equal amount of data from each class for training and validation purposes. This approach aimed to maintain a balanced ratio of one, thereby enhancing the model’s accuracy.

To ensure that the window contained the correct class of activation to be used to train the classification algorithm (bicep contraction, tricep contraction, or neutral arm state), it was considered that if the window contained 75% of the pulse width of the activation signal, then it was considered as such class; otherwise, it was regarded as a repetition of the neutral class. Finally, because in previous tests it was recorded that, for some participants, the sensor presented the behavior of, when being on for a time, the minimum value yielded by the sensor in steady state was considerably more significant than 0, it was decided to subtract this minimum component from the windows, taking into account that the pulse width of the contraction will always be less than the total time included in the window.

##### Feature Extraction and Feature Selection

Given the inherent complexity of sEMG signals and the available computational resources, a machine-learning model was employed for classification. Initially, 18 features were extracted from each sEMG channel, including commonly used time and frequency domain features for sEMG classification models [38,46,52,53]. However, not all of these features were incorporated into the final model; a thorough evaluation was conducted to select the most relevant features for improved classification accuracy, speed, and efficiency.

The features, numbered from 1 to 18, were as follows: The first 12 time-domain features included mean, standard deviation, root mean square value, skewness, kurtosis, distance from the maximum peak to the midpoint of the wavelength, distance from the top peak to the end of the wavelength, wavelength, zero-crossing rate, RMS slope, activity, mobility, and complexity. The final six frequency-domain features comprised the maximum power frequency, total power, mean frequency, frequency variance, and median power frequency.

These features, obtained for each sEMG channel, formed the basis for the subsequent selection and training of the classification model. The appropriate combination of these features could improve the classification system’s accuracy and robustness in identifying specific movements and gestures.

##### Classification Model and Feature Selection

Previous studies have shown the use of various machine learning and deep learning classification algorithms, such as k-nearest neighbor, support vector machine, principal component analysis, linear discriminant analysis, random forest, convolutional neural networks, dynamic time warping, and more. From the studies that performed real-time classification, SVM has shown one of the best performances overall [50,54,55,56,57].

SVM is a supervised machine learning algorithm primarily used for classification and regression tasks. The fundamental idea behind SVM is to find the optimal hyperplane that maximizes the margin between two classes, where the margin is defined as the distance between the hyperplane and the closest data points from each class, known as support vectors [58]. The chosen hyperplane acts as a decision boundary, classifying new instances based on which side of the boundary they fall on. In situations where the data are not linearly separable, SVM employs kernel functions to implicitly map the input data into a higher-dimensional space where a separating hyperplane can be found. Different kernel functions, such as linear, polynomial, radial basis function, and sigmoid, allow the SVM to model various nonlinear relationships between classes.

SVMs are inherently binary classifiers but can be effectively adapted for multi-class tasks. The prevalent adaptation method, especially in libraries like LIBSVM [59], is the “one-against-one” approach [60]. This technique constructs N(N − 1)/2 binary classifiers, each trained on a unique pair of classes from a dataset with N classes. During prediction, each classifier votes for one of its two classes when presented with a new instance. The class receiving the majority of votes across all classifiers is selected as the final classification. This approach capitalizes on SVM’s binary classification strengths and handles complexities in multi-class datasets, including potential class imbalances. By reframing the multi-class problem into multiple binary scenarios, SVMs maintain their robustness and accuracy in diverse classification contexts.

The sEMG signal classifier aims to distinguish bicep and tricep activities for prosthetic hand control. Various methods, such as neural networks, k-nearest neighbors, decision trees, linear and quadratic discriminant analysis, and kernel linear regression, were explored to find the most precise, efficient, and robust algorithm. The primary focus was to assess whether the multiclass classification SVM with the “one-against-one” approach, supported by prior studies, outperformed the other algorithms in this context. Using the MATLAB Classification Learner plug-in [61], the algorithms were semi-automatically trained with different parameters, hyperparameters, and kernels. This tool enabled model selection and automatic hyperparameter fitting to approximate an optimal model based on input and output data. Utilizing sEMG data from 2 participants, 75% of the collected repetitions were used for training, and the remaining 25% were reserved for validation. This comprehensive approach thoroughly evaluated each algorithm’s general performance, accuracy, and efficiency in classifying sEMG signals.

A sequential feature selection process was incorporated into the algorithm training. Initially, a detailed analysis and comparison of the information from various features were conducted. Given the high correlation between the two features, selecting both might contribute little new information and could lead to overfitting. Thus, a decision was made to choose only one of the strongly correlated characteristics. Examples included standard deviation, Hjorth activity centered on signal variance, average peak, maximum peak length, sum of frequency powers, and a signal´s average power.

Subsequently, three methods were applied for feature selection. First, the minimum correlation and maximum redundancy method was employed [62]. This method selects features based on two main criteria. The first, relevance, measures how well features discriminate between classes. The second, redundancy, measures the similarity between features. The goal is to select features with maximum relevance to the target class and, simultaneously, the minimum redundancy between them. The second method employed was neighbor component analysis [63]. This method seeks to optimize feature weighting to improve classification based on nearest neighbors. Unlike other methods that select a fixed subset of features, it assigns a weight to each feature, optimizing them so that instances of the same class move closer together and instances of different classes move farther apart in the weighted feature space. Finally, the third method employed was Relief-F [64]. This method evaluates the relevance of features based on their ability to distinguish between close instances of different classes. For each instance, Relief-F considers its nearest neighbor from the same class and its nearest neighbor from another class, considering a feature relevant if it has significantly different values for these two neighbors. These three feature selection methods aim to select distinctive and relevant features, reducing dimensionality to enhance classification efficiency while avoiding redundancy.

The sEMG classification algorithms, abbreviations used in subsequent tables, their configurations, and parameters are presented in Table 4. These algorithms underwent training using the individual and joint data of the participants. An initial comparison between models designed to classify three classes (bicep, tricep, and neutral) and those for four classes (bicep, tricep, isometric bicep and tricep contraction, and neutral) revealed a consistent trend. The three-class models exhibited a significantly higher accuracy, surpassing the four-class models by over 10% in overall accuracy (*p* < 0.01) based on a Kruskal–Wallis test and Tukey–Kramer post hoc test. This discrepancy was expected due to the limited control capacity of the upper arm muscles in amputees. Consequently, a decision was made to employ the three-class classification approach exclusively for gesture recognition and prosthesis control since a 10% difference in accuracy is highly undesirable for real-time implementation and since the same number of hand gestures can be performed with 3 class classification. Under this paradigm, bicep contraction controlled hand opening and closing, tricep contraction changed the gesture, and the neutral state maintained the hand’s position. Following this, the details of the three-class classification models will be discussed.

Table 5 displays 5-fold cross-validation accuracies for models trained with three classes using pooled data from both participants, as outlined in Table 4. The configurations involved varying sets of features, ranging from 18 to 5 features per channel. Notably, using five features did not show a significant statistical difference compared to using more (*p* > 0.1), as determined using a Kruskal–Wallis test. Among the top-performing algorithms utilizing a 5-feature set were cubic SVM (95.7%), fine decision tree (95.8%), fine k-nearest neighbors (97.6%), and wide neural network (97.5%). These outcomes resulted from 5-fold cross-validation and MATLAB Classification Learner automatic optimization, highlighting the potential for further improvement through additional fine-tuning.

**Figure 16:**
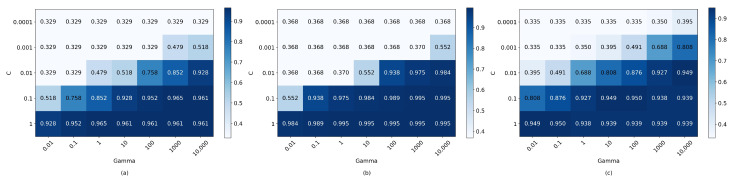
Cubic SVM 5-fold cross-validation accuracy heatmaps based on C and Gamma hyperparameters: (**a**) LV, (**b**) OB, and (**c**) LV and OB combined data.

**Figure 17:**
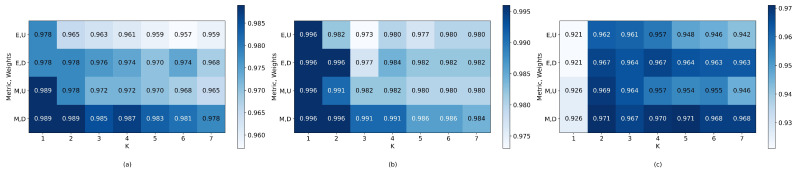
k-nearest neighbors 5-fold cross-validation accuracy heatmaps based on the number of neighbors, weight function (U for Uniform, D for Distance), and distance metric (E for Euler, M for Manhattan): (**a**) LV, (**b**) OB, and (**c**) LV and OB combined data.

**Figure 18:**
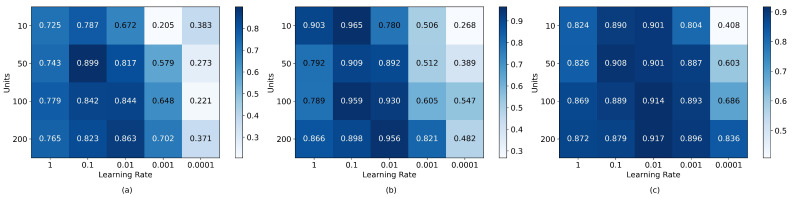
Neural network 5-fold cross-validation accuracy heatmaps with respect to single layer units and Adam optimizer learning rate: (**a**) LV, (**b**) OB, (**c**) LV and OB combined data.

Consequently, heatmaps were created to assess various parameters and hyperparameters for cubic SVM, k-nearest neighbors, and neural networks. Cubic SVM focused on the hyperparameters C and Gamma; k-nearest neighbors on the number of neighbors, weight function, and distance metric; and neural networks on the number of units in a single layer and Adam optimizer learning rates. Considering the unique sEMG signal characteristics and significant variance between individuals [48], algorithms were optimized, post-feature optimization, using sEMG datasets with only five features: individual and combined participant data. Figure 16, Figure 17 and Figure 18 display the resultant heatmaps for these three algorithms and datasets, showcasing the 5-fold cross-validation accuracy relative to the chosen parameters and hyperparameters.

**Figure 19:**
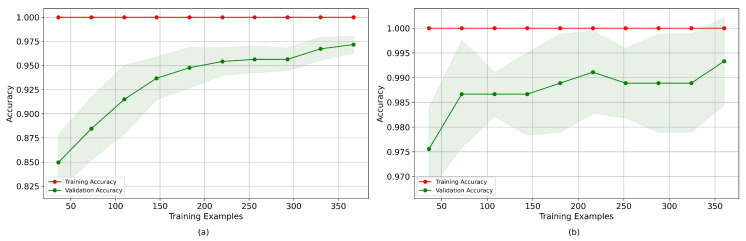
SVM training and validation accuracy curves for training examples with 5 features: (**a**) LV’s data, (**b**) OB’s data.

**Figure 20:**
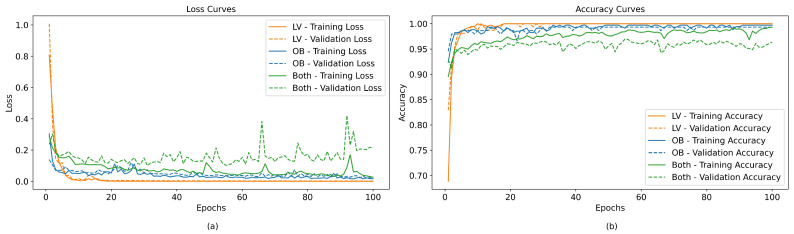
Training and validation metrics of a wide neural network trained with LV, OB, and combined datasets: (**a**) loss curves, (**b**) accuracy curves. Parameters: learning rate = 0.1, units = 100, epochs = 100, hidden units activation = ‘relu’, output activation = ‘softmax’, loss = ‘categorical crossentropy’.

Furthermore, an analysis was performed of the influence of the number of samples on the accuracy of the 5-feature per channel Cubic SVM-based models for both participants using 5-fold cross-validation. Figure 19 shows that for participant LV, an accuracy of approximately 82.5% was achieved with less than 50 samples. As the number of samples increased, the accuracy increased significantly to 98%, with a reasonably low variance for more than 300 samples. In the case of participant OB, less than 50 samples were necessary to reach an accuracy greater than 97.5%, and then this converged to 99% with more samples. From this result, it might be expected that neural network-based models converged faster to OB’s data. However, when analyzing the effect of the dataset on the convergence time of the loss and accuracy metrics of the neural networks-based models, as shown in Figure 20, it was observed that, in the case of LV’s data, the network converged in less than 20 epochs, while for OB’s data, it did not converge until after 110 epochs; while in the case of the model with joint data, the loss curve and accuracy of the validation data began to worsen after 60 epochs.

### 2.4. Prosthesis Functional Assessment Protocol

Prosthesis validation with volunteers was performed using the AM-ULA test developed by Resnik [65]. The AM-ULA test is a clinical instrument that assesses the ability of a person with upper limb amputation to perform functional activities of daily living using their prosthesis. In this assessment, volunteers are evaluated according to 5 categories: degree of performance, speed of performance of all activities, quality of movement, skill in using the prosthesis, and independence. Each class is graded on a scale of 0–4 (from not able to excellent). To obtain the total score, the scores of all categories are added together, averaged, and then multiplied by 10.

Resnik suggested that the protocol can be adjusted based on the specific prosthetic device being assessed and the equipment available for the tasks. The activities performed in this study are defined in Table 6.

Participants were instructed to perform these tasks to the extent of their capabilities. No specific time limit was imposed for the completion of each task. Table 7 shows the criteria used to assign a score to the tasks based on the following:

1. Completion of sub-task: Grades the extent of completion of all sub-tasks of the activity. A score of 0 points signifies that the user was unable to complete the sub-task;
2. Speed of completion of the entire activity. Grades the speed of task performance compared to performance with a sound limb;
3. Movement quality: Grades the amount of awkwardness or compensatory movements resulting in/from lack of prepositioning, device limitations, lack of skilled use, or any other reason;
4. Skillfulness of prosthetic use. Grades the type of use (no active use, use as a stabilizer, assist, or prime mover) and control voluntary grip functions;
5. Independence. Grades the use of assistive devices or adaptive equipment.

Two volunteers with transhumeral amputation were invited to perform the modified AM-ULA test: OB and LV. They were instructed to wear their customized LIBRA NeuroLimb prostheses with the sEMG and EEG sensor arrays. In this case, the battery embedded in the prosthesis was used to power it, and a laptop was used to perform the necessary signal processing and wirelessly control the prosthesis.

The procedure for each task unfolded as follows: Initially, the prosthesis started and remained in a ‘standby’ mode, indicated by a red LED on the prosthetic palm. In this state, any muscular movements made by the volunteer did not activate the hand’s motors. Activation only occurred when the "ON" mental command switched the prosthesis into an ‘activated’ mode, indicated by a green LED on the palm. At this point, muscle activity began to be taken into account for hand gesture control. In the ‘ON’ state, when the volunteer flexed their triceps muscle, this determined the type of gesture to be executed. Each flexion triggered a transition to a different pair of gestures, cycling through a set of three pairs of gestures (cylindrical grip with the open thumb, cylindrical grip with the closed thumb, and pinch grip) before returning to the first one. In contrast, based on the current hand gesture, the biceps muscle was used to open or close the hand. This meant that one flexion would close the hand, a subsequent bicep flexion would open it, and another flexion would close it again.

To execute a sequence such as picking up a cup of water and bringing it to the mouth, both volunteers followed a series of steps:

a. Mental command: switching from deactivated to activated mode.
b. Muscular command: selection of gesture for cylindrical type grip.
c. Bring the extremity of their arm closer so that the hand is positioned around the cup.
d. Muscular command: first flexion of the bicep. This flexion activates the grip.
e. Mental command: switch from activated mode to deactivated mode. The grasping gesture is maintained.
f. Activate the passive DOF at the wrist and elbow to achieve the desired position.
g. Bring the arm towards the face to drink from the cup.
h. Place the empty cup back on the table by the activating passive DOF at the wrist and elbow.
i. Mental command: switch from deactivated mode to activated mode.
j. Muscular command: second flexion of the biceps. This flexion activates the extension of the fingers.
k. Mental command: switch to deactivated mode.

Before the assessment sequence for each exercise, each volunteer performed up to two trials of the activities listed in the table to familiarize themselves with the activity. After these trials, the examiners carried out the evaluation.

## 3. Results

### 3.1. Mechatronic Prosthetic Performance

Prosthetic performance for force, power consumption, and execution time was evaluated. This section details the sensor configuration, sensor calibration, data acquisition methods, data analysis, and results.

The sensor utilized for quantifying the force applied by the fingers was a Sparkfun Robotic Finger Sensor V2. Calibration involved taking twenty-eight readings from objects with known masses (up to 6 kg) and establishing a linear model curve in MATLAB (R-squared ≈ 0.998) to correlate digital pressure readings with kilograms.

Force measurements were conducted to ensure the accuracy of the results. The sensor was secured to a stable surface, and the prosthetic hand gestured to reach maximum finger flexion. The external force was gradually applied until the finger moved, determining the maximum force it could exert. This process was repeated for the middle finger and thumb, accounting for lateral movement.

Calculation of current consumption during the activation of each finger was conducted by measuring the current drawn from the power source. This value included the energy consumed by the servo motors with load or movement, resting servomotors, and any other electronic components connected to the same power source. Considering 6 V servo motors, power and current consumption calculations were performed. Table 8 presents the results of maximum force measurement and the corresponding maximum current consumption for the index, middle, and thumb fingers.

Finger travel time, from the full extension to its full flexion position, is a critical parameter for assessing the speed and efficiency of a prosthesis. The predefined usage times for the prosthesis were 2 s for the index and middle fingers and 1.2 s for the thumb; however, the travel speed can be customized according to the user’s preference. To understand the mechanical capabilities of the motor and finger mechanism, a test was conducted to determine the minimum travel times. In this experiment, an Adafruit BNO055 orientation sensor was fixed to the tip of each finger. The fingers underwent two sets of 20 repetitions each, moving from their initial positions. Data were collected at a sampling frequency 200 Hz, ensuring a maximum time measurement error of 0.01 s due to the motion sensor’s update rate. Subsequently, time vs. sensor digital reading plots were generated, and visual analysis of the graphs was performed to determine time differences. Table 9 presents the calculated mean, minimum, maximum, and standard deviation time in seconds.

**Figure 21:**
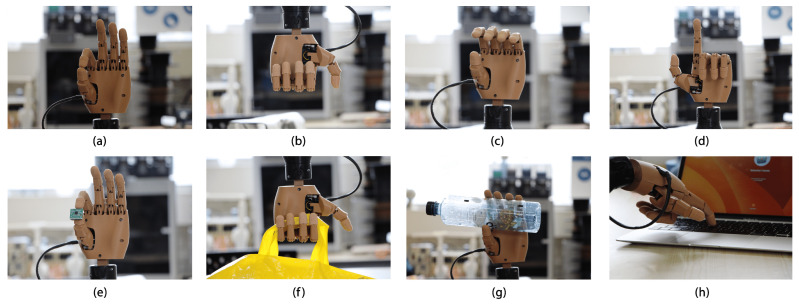
Prosthesis gestures: (**a**) cylindrical gesture, (**b**) hook gesture, (**c**) cylindrical gesture, (**d**) finger-pointing gesture, (**e**) cylindrical grip, (**f**) hook grip, (**g**) cylindrical grip, (**h**) finger-pressing gesture.

Finally, validation was completed for the four proposed gestures for the prosthesis. Performing each gesture in three positions—forearm in a vertical position with the hand facing upwards, forearm in a horizontal position with the handheld horizontally, and forearm in a vertical position with the hand facing downwards—was conducted to achieve validation. Figure 21 also displays the prosthesis gestures without objects in a vertical position. Additionally, each gesture was executed by grasping an object: cylindrical grip with a mini driver, hook grip with a plastic bag weighing approximately 50 g, cylindrical grip with an empty plastic bottle, and finger-pressing gesture for pressing keys on a laptop.

### 3.2. Offline Hybrid Classification System Performance

This study proposed using a hybrid control system that integrates sEMG and EEG signal classification for active control of the LIBRA NeuroLimb transhumeral prosthesis. For this purpose, different models were trained to classify the arm activity for the activation and change of gestures of the prosthetic hand, and the EMOTIV BCI application was used for the training of mental commands through the use of its integrated artificial intelligence model for the activation of the gesture classifier, avoiding unwanted predictions and therefore unintentional movements.

Multiple models were evaluated using the data from both participants with different numbers of features, from 18 to 5 per channel as shown in Table 5. It was corroborated that there was no statistically significant difference in accuracy when more than five features were used (*p* < 0.05). Subsequently, the three best-performing models (cubic SVM, fine k-nearest neighbors, and wide neural network) were chosen for optimization based on three datasets: two corresponding to the individual data of each participant, and one with pooled data. As detailed previously, the objective was to classify three distinct upper arm movements: bicep contraction, tricep contraction, and arm neutral state. Offline performance metrics for the models were derived from 25% of the total data, while the remaining 75% was utilized for training. Moreover, it was ensured that the imbalance ratio was one by using the same amount of data per class for training and validation, increasing the model’s accuracy by avoiding pushing the decision boundary towards the minority classes [66].

The five-fold cross-validation accuracies resulting from the optimization of parameters and hyper-parameters can be seen in Figure 16, Figure 17 and Figure 18. Table 10 shows the accuracy, precision, recall, f1-score, sensitivity, specificity, and predictions per second of the best-optimized models for each dataset. It is asserted that the algorithms based on k-nearest neighbors obtained the best results in terms of accuracy and were superior to the neural network and SVM-based models concerning this metric (*p* < 0.05), with an accuracy difference of 2% for the best model; however, the SVM-based models had a much shorter prediction time, surpassing both k-nearest neighbors and neural networks-based models (*p* < 0.01). Consequently, the optimal accuracy and speed models were chosen for integration into the real-time system, enabling the selection of the most effective model in this operational context.

**Figure 22:**
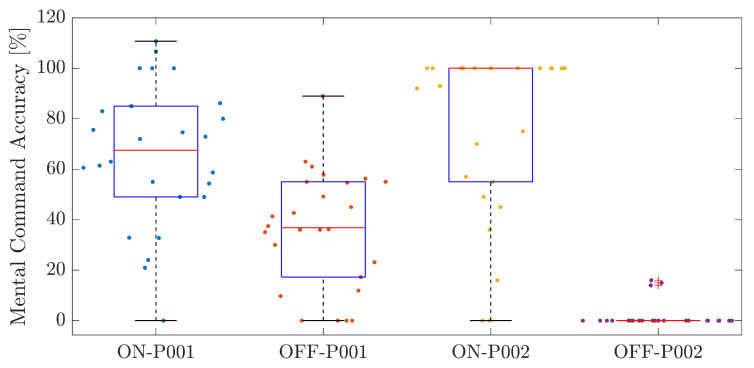
EEG classification algorithm accuracy across participants (LV and OB) and commands (ON and OFF) per repetition. P001 and P002 refer to LV and OB, respectively.

Mental commands were used to activate and deactivate the arm gesture classifier. For this purpose, an electroencephalography-based brain–computer interface was employed using an EMOTIV EPOC X device and EMOTIV BCI interface. The results regarding the training of the mental command classifier model with this application were suboptimal. Participant LV achieved accuracy scores of 65.70 ± 28.23% for the ‘ON’ mental command and 36.25 ± 23.20% for the ‘OFF’ mental command. In contrast, participant OB recorded 76.46 ± 33.40% for the ‘mental on’ command but a markedly low 1.73 ± 4.89% for the ‘mental off’ command. Given the necessity for accuracy peaks exceeding 75% to activate a mental command within the control system effectively, these outcomes suggest significant challenges in consistently achieving this threshold. This was particularly evident for the ‘mental off’ command for both participants, with OB’s near-zero mean accuracy of 1.73% being especially concerning. Figure 22 visually represents the distribution of accuracy outcomes for both commands across the participants.

### 3.3. Experimental Validation Results

Two volunteers with transhumeral amputation, OB and LV, performed the modified AM-ULA test described in Section 2.4 to assess the ability of two random users to perform functional activities of daily living using the LIBRA Neuro prosthesis.

**Figure 23:**
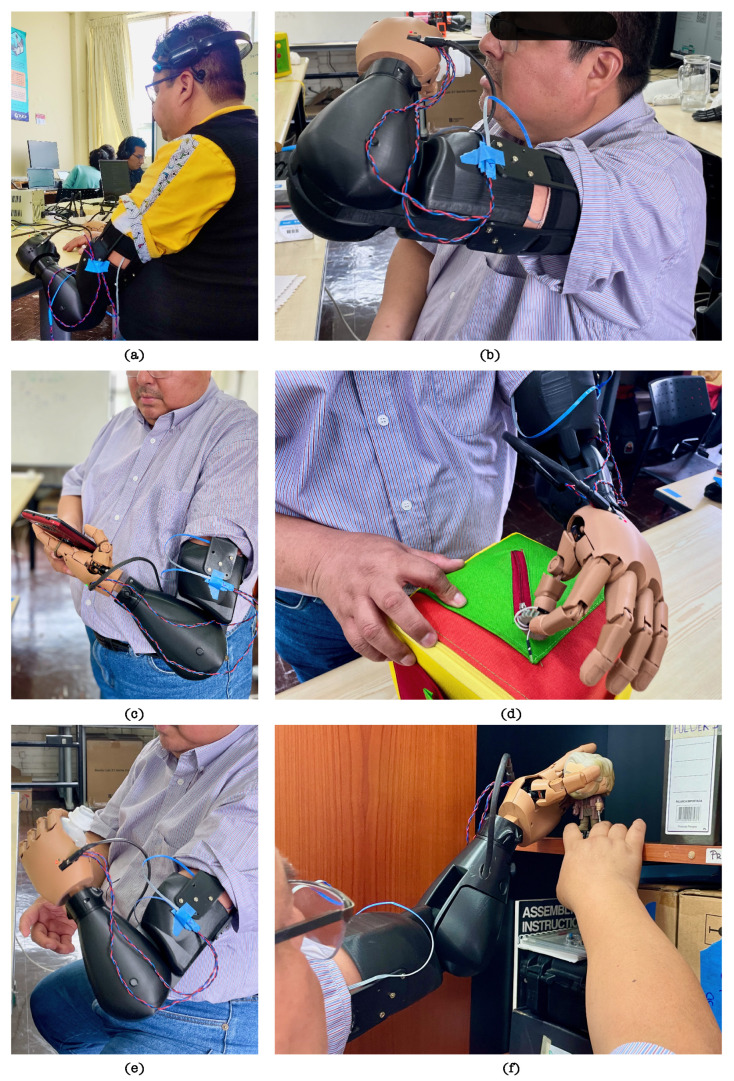
Volunteer OB performing training and AM-ULA sub-tasks: (**a**) Training with EEG sensors. (**b**) Simulated drinking. (**c**) Holding his cell phone. (**d**) Zipping sub-task. (**e**) Holding a cup of water. (**f**) Reaching an object on a shell.

**Figure 24:**
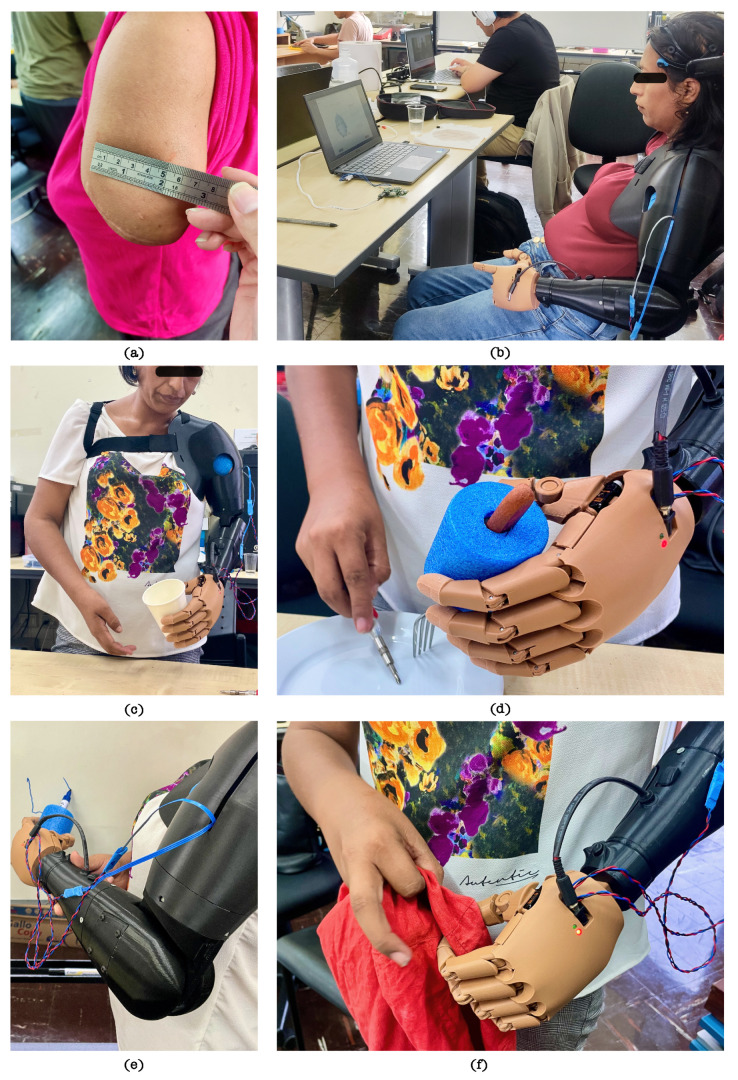
Volunteer LV performing training and AM-ULA sub-tasks: (**a**) sEMG sensors placement measure on amputated section. (**b**) Training with EEG sensors. (**c**) holding a cup of water. (**d**) Using cutlery. (**e**) Writing initials on a board. (**f**) Folding a piece of cloth.

As previously established, AM-ULA is a clinical instrument that measures the ability of a person with upper limb amputation to perform daily functional activities using a prosthesis. Each item is scored on a scale of 0–4 (incapable to excellent). To obtain the total score, the scores of all the items are added, and then the mean is calculated and multiplied by 10. In a perfect execution of the modified AM-ULA test, where all aspects of each subtask receive the highest possible rating, a total score of 480 can be achieved. In the practical application of this test, volunteer OB attained a score of 222, whereas volunteer LV secured a score of 144. Task scores from each participant are shown in Table 11. Photographs of the sessions with both volunteers are shown in Figure 23 and Figure 24.

In the case of volunteer OB, the highest scoring task was ‘drinking water from a cup’, followed by ‘running the zipper-skill cube’ and ‘writing initials’, activities that represent skillfulness in both gross and refined types of grasps. For volunteer LV, the highest scoring sub-tasks were those of grasping type: ‘fold a piece of clothing’ followed by ‘pour liquid into a cup’. Furthermore, volunteer OB completed all the activities proposed in the trials. In contrast, volunteer LV needed help to complete 5 of the 12 proposed activities.

## 4. Discussion

This study presents the design and development of the LIBRA NeuroLimb transhumeral prosthesis, featuring a hybrid control system based on the classification of muscle and neural signals and designed to be customizable and accessible for use in developing countries. As highlighted in the introduction, the prevalent commercial proposals originate from the United States and Germany, which are developed nations where private or public insurance primarily covers the expenses of prosthetics, estimated between USD 20,000 and 100,000. These costs are beyond reach in economies like Peru, where the minimum wage is approximately USD 320 monthly. Furthermore, ad hoc customization is unavailable for each user in commercial models, as they typically come in predetermined sizes. Moreover, the control system is predominantly mechanical or electromyographic reading in almost all cases.

The prosthesis is designed to be customizable and functional, tailored to the user’s level of amputation and residual arm functionality. Control is achieved by activating the residual arm’s biceps and triceps and mental commands, i.e., classifying sEMG and EEG signals. The arm features three active and four passive DOF with three primary gestures: a cylindrical grip with open and closed thumb and a pinch between the thumb and index finger. Finally, the design focused on functionality, user acceptability, and comfort, reflecting a user-centered design approach.

Given the need for a high level of customization, digital manufacturing technologies were pivotal. The process began with 3D scanning of the volunteers’ limbs, followed by creation of digital models of the amputations. These models informed the design of the prostheses, which were then fabricated using 3D printing. This method allowed for adjustments in interior density, enhancing the strength in load-bearing parts, while reducing weight in giant shell and cover components. A modular design facilitated the development and validation of this proof-of-concept design.

Signals from the bicep and triceps were instrumental in controlling the prosthetic hand’s gestures, specifically their selection, opening, and closing. For this purpose, an sEMG signal classifier was developed to classify various activation types for transhumeral amputees’ remnant arm. Different algorithms were trained to select the most optimal one for employment in the real-time system. It was found that the algorithms that best fit the data were cubic SVM, fine k-nearest neighbors, and wide neural networks. Four classes were initially expected to be classified, considering the user’s ability to perform bicep and tricep individual and joint isometric contractions. However, the isometric contractions were confused for tricep contractions, reducing classification accuracy by more than 10% (*p* < 0.01). Therefore, it was decided to reduce the number of classes to three. Alongside, after a systematic feature selection process, it was validated that it was equally accurate to use five features compared to using more (*p* < 0.05), significantly speeding up feature extraction and model classification.

A parameter and hyperparameter sweep for the three models above was performed, to select the optimal algorithm for three different datasets: each participant, and the joint data of both. As shown in Table 10, the k-nearest neighbor classifier obtained the best accuracy for all datasets, with a maximum accuracy of 99.8% and a difference of up to 2.1% accuracy for the case of LV’s data concerning the other models. In contrast, the cubic SVM model, despite achieving a maximum accuracy of 99.2% for the case of OB’s data, could perform up to five times more predictions than the algorithm based on k-nearest neighbors. Thus, it was decided to integrate and test the best-performing algorithms in the real-time system to evaluate their performance, given the significant difference between offline performance in training scenarios and performance in a test scenario where users were required to execute unexpected movements, grasp and move objects, among other variations.

The sEMG classification models achieved 99.8% accuracy, aligning and, in some cases, surpassing the state-of-the-art models. However, it should be noted that in [38,53,67], classification was performed on seven, eight, and nine classes, respectively, achieving accuracies of 90%, 88.5%, and 93%. In contrast, the current model required the classification of only three classes, which could suggest an intuitive increase in accuracy due to having fewer categories. Nevertheless, it is crucial to consider that the studies mentioned above utilized five, six, and six sEMG channels, respectively, whereas our study employed only two. Consequently, one can only conclusively attribute an increase in accuracy to the reduction in class number by accounting for the lower dimensionality of the data involved. Additionally, the level and type of amputation notably influenced the model’s precision.

Table 12 offers a comparative analysis of the LIBRA NeuroLIMB sEMG classification algorithm against other studies in the field. It showcases the algorithm’s notable accuracy of 99.8% for identifying three gestures using just two channels. This notable accuracy contrasts with other studies that classified a broader range of gestures but required more electrodes and generally achieved lower accuracies. Hence, a straightforward comparison of accuracies is only entirely appropriate when considering these aspects, including the reliance of other studies on offline validations, in contrast to the real-world applicability of the LIBRA NeuroLIMB approach. Therefore, while this study, in light of its inherent limitations, refrains from asserting the superiority of the control system when compared to the state of the art, it serves as a proof of concept that integrates the classification of sEMG and EEG signals within an architecture based on parallel and concurrent tasks. This contribution can enhance classification algorithms, even using more complex models, without compromising the classification time or control of upper limb prostheses. The significance of this work lies in its potential to advance contributions to upper limb prosthetic control systems. Although inaccurate, this approach is promising for future enhancements and the development of cost-effective prostheses, especially in resource-limited settings like Peru. This study emphasizes the growth potential, focusing on developing affordable and efficient prosthetic control systems.

In addition, Figure 19 shows that LV, with a proximal amputation and less remaining muscle, needed more data to improve accuracy. In contrast, OB, with a distal amputation and more retained musculature, reached over 97% accuracy with fewer than 50 samples. These findings suggest that even with amputations that yield less than ideal signals, it is possible to attain high accuracy by employing robust feature extraction, selection, and model optimization processes, including parameter and hyperparameter tuning, coupled with a sufficient volume of data.

EEG signals toggled the sEMG classification algorithm ON and OFF, ensuring the system only interprets intentional hand movements. The EMOTIV EPOC X and EMOTIV BCI applications were used to train and predict mental commands. However, EEG signal classification yielded a moderate accuracy, necessitating a single mental command to reliably control the sEMG classifier’s state. This approach proved effective and required minimal user adaptation. Nevertheless, the EEG classification model exhibited moderate mean accuracies, with LV’s commands recognized at 65% and 36%, and the second volunteer’s at 76% and 1.73%. To enhance control stability and success rates, it was necessary to simplify the system to respond to a single mental command to toggle the sEMG classifier’s state on and off. This simplification did not compromise the model’s effectiveness, as simultaneous execution of multiple mental commands was not required, due to the sequential nature of state changes. However, it did necessitate users to adapt to utilizing a single mental command for activation and deactivation, a process which, while necessary, was quickly adopted by the users.

Compared with other prosthetic developments, the LIBRA NeuroLimb demonstrated adequate grasping force, as highlighted in [68]. However, some prostheses with metallic structures, referenced in [19,31,69], exhibit force capabilities exceeding 67 N, compared to ours at 21 N. The LIBRA NeuroLimb’s force capacity could be enhanced by incorporating more robust materials in its internal structure and mechanisms. Redesign of the activation link mechanism is necessary, as wear on the link joint over time delays the finger group actuation system. Regarding speed, while the prostheses mentioned above complete object grasping in a minimum of 2.8 s, the LIBRA NeuroLimb achieves closure in as little as 0.2 s. Future work will consider using robust thermoplastics for the components that bear higher mechanical loads, to increase the load-bearing capacity and extend the prosthesis’s lifespan.

The prosthesis’ performance was validated with the modified AM-ULA test to assess the ability of users to perform functional activities of daily living using the prosthesis, in addition to its technical performance and usability. Two volunteers, LV with a distal transhumeral amputation and OB with a proximal transhumeral amputation, both resulting from accident-related trauma, participated in the training and validation tests.

In the practical application of this test, volunteer OB scored 222, whereas volunteer LV secured a score of 144. Variations in AM-ULA scores can stem from the level of amputation, muscle fatigue, and sensor displacement. LV’s proximal amputation (close to the shoulder) limited her range of motion, preventing the completion of specific AM-ULA tasks, especially those requiring arm elevation above shoulder height. Conversely, OB, with his elbow-level amputation, completed all tasks without significant difficulties, due to greater arm mobility. Compared with other prostheses that have undergone AM-ULA evaluation, ref. [6] states that the instrument is intended to allot relatively low scores. This is due to its penalization of situations where the task has not been fully completed, resulting in a score of zero. In 2013, the instrument was applied to 46 volunteers, and those with myoelectric transhumeral prostheses achieved scores ranging from 140–160. The volunteers had a mean age of 45.8 ± 16.5. In contrast, in 2020 [70], 112 volunteers were studied, and those with myoelectric transhumeral prostheses achieved scores averaging 110. The mean age of the volunteers in this study was 56.7.

As the volunteers progressed through the tasks, muscle fatigue became evident, particularly in the latter stages, affecting the selection of hand gestures and overall task execution. This fatigue impacted the time, dexterity, and movement quality, as reflected in the task scores. Despite the prostheses being tailored to each user’s limb weight, future iterations will aim for lighter models, to minimize fatigue. Notably, both volunteers were already accustomed to button-activated prostheses but needed to gain experience with sensor-controlled devices. To address this, future protocols will include pre-prosthetic training with occupational therapists, focusing on muscle activation and signal interpretation, to enhance preparedness and reduce muscle fatigue.

Post-evaluation, sensor displacement and sweat accumulation were noted, which was anticipated due to the sensors’ placement in a high-mobility zone and the PLA prosthetic socket’s material. Future designs will focus on minimizing sensor displacement and developing a breathable, carbon fiber-based socket to reduce sweating without sacrificing comfort, durability, and rigidity. Additionally, internal cavities will be considered to position and conceal sEMG sensor wiring as part of the internal components.

Variations in results also stemmed from the user’s physiology, environmental interference, and limitations in the signal acquisition system and classification model. Factors such as body movement, changes in arm position, force exertion, and variations in arm load can negatively impact classification accuracy [52,71,72]. These factors can distort the acquired signals, add noise, or alter signal frequency characteristics. Although preprocessing can attenuate some effects, significant changes in signal characteristics may require a more robust classifier model and more extensive training data.

In practice, it was observed that despite high offline accuracy, the model sometimes faltered under real-life conditions involving prosthetic load, body, and arm movement. For instance, when volunteer OB attempted to pick up an object from a shelf above his head, the model erroneously detected bicep contractions due to the combined weight of the prosthesis and the object. This false reading occurred because the model confused arm elevation with active contraction, highlighting the model’s vulnerability to untrained or abnormal movements and potential overfitting.

The hybrid control system of the prosthetic, while demonstrating exemplary performance in offline sEMG evaluations, faced challenges when implemented in real-time, real-life situations. These challenges stemmed, not from computational or logical control flow demands, but from limitations in the sEMG and EEG classification algorithms. With only two sEMG electrodes placed on the residual bicep and tricep of the user with a transhumeral amputation, the number of distinguishable classes was constrained due to the possible independent and combined muscle activations. Consequently, this led to the use of only three classes and necessitated sequential movements for comprehensive prosthetic hand control, which could increase the task completion time, especially in the event of misclassification. Despite achieving over 99% accuracy offline, the sEMG algorithm misclassified actions during physical tasks, such as when the user’s arm was under significant load or elevated, like reaching for an object on a shelf. The algorithm’s training did not include these scenarios, indicating potential overfitting to training data. Efforts were made to avoid classification errors from unbalanced data by ensuring equal data amounts per class during training and validation.

To address these issues, enhance model robustness, and prevent overfitting, future work should include diverse training scenarios with varying prosthetic arm positions, forces, and loads, to improve model robustness and prevent overfitting [52]. If feasible, the number of sEMG electrodes should be increased to capture more signals from the bicep and tricep and adjacent areas like the shoulder and back, to eliminate the need for sequential movements and allow for more complex muscular activations since, as seen in previous work, expanding the electrode array to record surrounding muscles, in conjunction with phantom arm movements, may yield better results [53]. This approach could increase DOF and enable parallel movement execution rather than sequential, leading to a more responsive and accurate system. Moreover, this could avert the need to limit the sEMG classifier to three classes, allowing for distinguishing more complex muscle activations. More sophisticated deep learning models like convolutional or recurrent neural networks could analyze sEMG signals directly using time or frequency, with minimal preprocessing.

Regarding the EEG signal classifier, there is room for improvement in the achieved accuracy. While LV’s mental commands were classified with an average accuracy of 65% and 36%, OB’s first command averaged 76% accuracy, and the second was barely recognized, averaging 1.73%. This led to the decision to use a single mental command to turn the sEMG signal classification on and off, better recognize the user’s intent, and avoid misclassifications due to noise or involuntary movements. These limitations could have been due to EEG electrode displacement, mental fatigue, or the user’s concentration capacity. LV was entirely focused on the first mental command but less on the second, whereas OB, despite being concentrated, could not activate the second command. The EMOTIV BCI software (version 3.6.5.262) was used for classifying mental commands, not a customized algorithm, primarily due to the additional cost of obtaining a license for raw data acquisition with the EMOTIV EPOCx, which exceeds USD 1000 annually.

Future work should prioritize implementing a proprietary EEG signal classification algorithm, validating if a higher precision in EEG classification is achievable. If improvements are minimal, alternative approaches should be considered, or a greater emphasis should be placed on optimizing the sEMG classification model to function independently and accurately.

The prosthesis presented in this work is designed and considered replicable through applying technologies that are accessible in developing countries by leveraging accessible technologies. The design of the prosthesis is parametric, as it is intended for distal, middle, and proximal transhumeral amputations for both male and female physiognomies. The prototype stands as a proof of concept (TRL-3), enabling the researchers to validate their hypotheses related to the control design and to iterate on the design proposal. Likewise, reading brain commands in real time was possible for both the training and validation tests, due to the modular design that incorporates a laptop and a brain–computer interface, acquired for research purposes.

The activation–deactivation system of the prosthesis with brain commands proved successful both in the training and the AM-ULA testing phases. However, the costs of an accessible prosthesis implementation are too expensive. In this sense, in future implementations of the prosthetic model that bring development closer to a TRL-7 or TRL-8 level, other means of activation might be used, such as voice recognition or electromyographic reading of the muscles of another section of the body. These alternatives promise to retain the effective activation–deactivation mechanism demonstrated by the brain–computer interface but to reduce costs and enhance the accessibility of the prosthesis significantly.

## 5. Conclusions

This work presented the design, development, and validation of the LIBRA NeuroLimb, an accessible transhumeral prosthesis designed for the nuanced requirements of people with upper limb amputation in developing regions. The prosthesis features a parametric design adaptable to mid, distal, and proximal amputations, accommodating both male and female users. Its production leverages digital fabrication techniques, which ensure the prosthesis’s scalability and accessibility. The prosthesis exhibits three active and four passive DOF. It incorporates a hybrid sEMG and EEG-based control system that demonstrated high accuracy (99.8% for sEMG signal classification) and functional utility in preliminary tests after a rigorous optimization process via algorithm selection, feature selection, and hyperparameter tuning. Despite the EEG component’s lower-than-desired accuracy, strategic simplification to a single mental command made the system viable for real-time control. The prosthesis was validated using the ‘Activities Measure for Upper Limb Amputees’ (AM-ULA) test on individuals with varying levels of transhumeral amputation, resulting in scores of 222 and 144. These results promise to improve the users’ ability to perform daily activities. Future research will enhance mechanical strength through material upgrades, increase the number of active DOF, and refine the control system to accommodate real-world challenges better. This study’s findings set the stage for continued advancements in prosthetic solutions that are technologically sophisticated and economically feasible in developing countries.

## Figures and Tables

**Table 1:** Comparison of commercial transhumeral models in 2023.

Aspect	LUKE Arm [24]	Utah Arm [23]	Dynamic Arm [21]	ErgoArm Electronic Plus [22]
Country of origin	US	US	Germany	Germany
Cost (USD)	50 K–100 K	20 K	76 K	27 K
Control mode	sEMG	sEMG	sEMG	sEMG or buttons
Compatibility	-	Ottobock	Ottobock, Bebionic	Ottobock, Bebionic
Active DOF	3	3	3	1
Weight	3.5 kg	913 g *	1 kg *	550–710 g *
Battery	Li-ion	Ni-MH	Li-ion	-
Customization	Socket only **	Socket only **	Socket only **	Socket only **

* Not considering hand weight. ** Standard sizes could be selected.

**Table 2:** Design requirements.

N°	Aspect	Requirements
1	Design	Compact and organic design using the anthropometry of a real user. Ensure materials that increase friction to prevent objects from slipping.
2	Kinematics	Maximum hand closing time of 1.5 s. Use self-locking mechanisms for passive systems.
3	Power	Ability to remove the power source. Not to exceed 10 mA of current in the sensors and 1000 mA in the actuators.
4	Materials	Resilient and corrosion-resistant. Hypoallergenic material for the prosthetic socket.
5	Signals	Power system operating at commercially available voltage levels. Non-invasive acquisition of physiological signals.
6	Safety	Prevent the ingress of liquids into the system. Manual disconnection of the actuator power source is possible.
7	Ergonomics	Maximum hand weight of 0.5 kg. Preventing excessive sweating.
8	Manufacturing	Selection of commercially available components.
9	Assembly	Prosthetic socket must avoid relative movements concerning the residual limb.

**Table 3:** Prosthesis motor specifications and comparison.

Characteristics	Power HD 1810MG	EMAX ES09MD II
Operating voltage (V)	6	6
Stall Torque (kgf-cm)	3.9	2.6
Speed (s/60°)	0.13	0.08
Weight (g)	15.8	14.8
Dimensions (mm)	22.8 × 12.0 × 29.4	23.0 × 12.0 × 24.5
Gear Type	Copper	Metal Gear

**Table 4:** Initial algorithm configurations and parameters for sEMG signal classification.

Algorithm	Abbreviation	Parameters
Linear SVM	L-SVM	Kernel function: Linear; Kernel scale: Automatic; Box constraint level: 1; Multiclass method: One-vs-One
Cubic SVM	C-SVM	Kernel function: Cubic; Kernel scale: Automatic; Box constraint level: 1; Multiclass method: One-vs-One
Medium Gaussian SVM	M-SVM	Kernel function: Gaussian; Kernel scale: 4.7; Box constraint level: 1; Multiclass method: One-vs-One
Fine Decision Tree	F-TREE	Maximum number of splits: 100; Split criterion: Gini’s diversity index; Surrogate decision splits: Off
Medium Decision Tree	M-TREE	Maximum number of splits: 20; Split criterion: Gini’s diversity index; Surrogate decision splits: Off
Linear Discriminant Analysis	LDA	Covariance structure: Full
Quadratic Discriminant Analysis	QDA	Covariance structure: Full
Fine KNN	F-KNN	Number of neighbors: 1; Distance metric: Euclidean; Distance weight: Equal
Medium KNN	M-KNN	Number of neighbors: 10; Distance metric: Euclidean; Distance weight: Equal
Cubic KNN	C-KNN	Number of neighbors: 10; Distance metric: Minkowski; Distance weight: Equal
Kernel Logistic Regression	KLR	Learner: Logistic Regression; Number of expansion dimensions: Auto; Regularization strength (Lambda): Auto; Kernel scale: Auto; Multiclass method: One-vs-One; Iteration limit: 1000
Narrow Neural Network	N-NN	Number of fully connected layers: 1; Layer size: 10; Activation: ReLu; Iteration limit: 1000; Regularization strength (Lambda): 0
Medium Neural Network	M-NN	Number of fully connected layers: 1; Layer size: 25; Activation: ReLu; Iteration limit: 1000; Regularization strength (Lambda): 0
Wide Neural Network	W-NN	Number of fully connected layers: 1; Layer size: 100; Activation: ReLu; Iteration limit: 1000; Regularization strength (Lambda): 0
Bilayered Neural Network	B-NN	Number of fully connected layers: 2; Layer sizes: 10; Activation: ReLu; Iteration limit: 1000; Regularization strength (Lambda): 0
Trilayered Neural Network	T-NN	Number of fully connected layers: 3; Layer sizes = 10; Activation: ReLu; Iteration limit: 1000; Regularization strength (Lambda): 0

**Table 5:** Five-fold cross-validation accuracies of the machine learning classification algorithms with different feature sets and configurations.

Features	L-SVM	C-SVM	M-SVM	F-TREE	M-TREE	LDA	QDA	F-KNN	M-KNN	C-KNN	KLR	N-NN	M-NN	W-NN	B-NN	T-NN
1-18	92.1	96.9	93.3	96.6	93.7	91.5	91.8	98.4	95.2	95	93.5	97.1	98.4	98.1	97.1	96.8
2, 4, 5, 7, 8, 12, 13, 15, 17	91.8	96.8	93.4	96.6	92.7	89.7	84.5	98.4	94.7	94.7	92.7	95.7	97.5	98.1	96.5	96.6
2, 4, 5, 7, 8, 12, 15, 17	91.9	96.8	93.6	96.5	92.8	89.6	83.7	98.3	94.7	94.6	92.7	95	97.2	98.4	95.7	95.9
2, 4, 7, 8, 12, 15, 17	91.5	96.8	93.5	96.6	92.5	89.3	87.7	98.3	94.7	94.6	92.3	95.1	96.8	97.7	96.1	96.3
2, 7, 8, 12, 15, 17	91.6	96.3	91.7	95.6	92.6	89.3	88.5	97.5	93.9	93.5	90.2	94.5	96.5	97.6	95.4	95.2
2, 7, 12, 15, 17	91.8	**95.7**	91.5	**95.8**	92.6	89.3	90.0	**97.6**	94.2	93.9	90.0	94.5	95.8	**97.5**	95.2	95.4
PROM	91.78	**96.55**	92.83	**96.28**	92.82	89.78	87.70	**98.08**	94.57	94.38	91.90	95.32	97.03	**97.90**	96.00	96.03
MAX	92.10	**96.90**	93.60	**96.60**	93.70	91.50	91.80	**98.40**	95.20	95.00	93.50	97.10	98.40	**98.40**	97.10	96.80

Refer to Table 4 for the complete names of the algorithms and detailed descriptions of their configurations and parameters. The accuracies of the best variants from the top-performing algorithms have been highlighted in bold.

**Table 6:** List of tasks and subtasks of the modified AM-ULA protocol.

N°	Sub-Tasks	Suggested Execution Order	N°	Sub-Tasks	Suggested Execution Order
1	Put ona shirt	(1) take the clothing (2) put it on the back (3) put the arms through (4) get comfortable	7	Use cutlery	(1) take the cutlery (2) bring the cutlery close to the mouth (3) move the cutlery away from the mouth (4) release
2	removeshirt	(1) take the garment (2) remove arms (3) place the garment on the table (4) loosen the garment	8	Pourliquid	(1) Take an empty glass (2) pour some water from another glass (3) leave glass that was emptied on table (4) drop the other glass that has been filled
3	Buttons	(1) get into position (2) push button through (3) pull the button (4) complete all buttons	9	Writeinitials	(1) take a marker (2) write your initials on the board (3) put the pen down
4	Zipper	(1) pick up the zipper (2) get comfortable (3) take the zipper to the other end (4) release	10	DialPhone	(1) take the cell phone (2) with the other hand dial a number (3) communicate on speaker phone (4) end call (5) leave the cell phone
5	Shoelace	(1) take a shoestring with prosthesis (2) take another shoestring with other arm (3) tie knot (4) tighten and loosen	11	FoldClothes	(1) take both ends (2) make a first fold (3) make a second fold (4) leave the folded garment on the table
6	Drinkwater	(1) hold the glass (2) bring the glass to the mouth (3) tilt the glass and simulate drinking (4) return glass to the table (5) release	12	Takeobjecton shelf	(1) raise arm (2) take the object (3) lower arm with object in hand

**Table 7:** Criteria used to assign ratings to each category of the modified AM-ULA protocol.

Grade	Speed of Completion	Movement Quality	Skillfulness of Prosthesis Use	Independence
Unable (0)	N/A	N/A	No prosthetic use	N/A
Poor (1)	Very slow to slow.	Very awkward, many compensatory movements.	Inappropriate choice of grip for the task (if choice is available). Loses grip multiple times during task, lack of proportional control (if available). Multiple unintentional activations of a control.	May or may not use an assistive device.
Fair (2)	Slow to Medium.	Some awkwardnessor compensatory movement.	Sub-optimal choice of grip for the task (if choice is available). Use of prosthesis to assist bimanual or prime mover unilateral activities. Loses grip once during the task. More than one attempt is needed to pre-position the object within grasp and more than minimal awkwardness in positioning the object. One incidence of unintentional activation of a control.	May or may not use an assistive device.
Good (3)	Medium-fast to normal.	Minimal to no awkwardness or compensatory movement.	Skilled use of prosthesis as an assist for bimanual activities or as a prime mover for unilateral activities. Quick and easy pre-positioning of the object within grasp. No unintentional loss of grip.	May or may not make use of the assistive device.
Excellent (4)	Equivalent to non-disabled.	Excellent movement quality, no awkwardness or compensatory movement.	No intentional loss of grip or unwanted movement. Optimal choice of grip for the task (if the choice is available).	May or may not make use of the assistive device.

N/A: not applicable.

**Table 8:** Force characterization results and maximum current consumption per finger.

Parameter	Index Finger	Middle Finger	Thumb
force (N)	21.26	11.12	16.22
current (A)	1.07	1.07	0.67

**Table 9:** Prosthetic fingers’ travel time in seconds using maximum velocity.

Value	Index Finger	Middle Finger	Thumb
mean	0.20	0.21	0.23
maximum	0.27	0.23	0.27
minimum	0.13	0.18	0.20
SD	0.04	0.02	0.03

**Table 10:** Performance of the top classification algorithms on individual and combined datasets.

Data	Algorithm	Parameters	Accuracy	Precision	Recall	F1-Score	Sensitivity	Specificity	Speed *
LV	Cubic SVM	C = 1000, Gamma = 0.1	0.977	0.979	0.974	0.976	0.964	0.989	22.9
OB		C ≥ 1, Gamma = 1	0.992	0.994	0.990	0.992	0.990	0.995	22.9
Both		C = 100, Gamma = 0.1	0.949	0.935	0.946	0.940	0.946	0.974	1.75
LV	k-Nearest Neighbors	k = 1, metric = manhattan, weights = distance	0.998	0.998	0.998	0.998	0.999	0.999	4.64
OB		k = 1, metric = manhattan, weights = distance	0.998	0.998	0.998	0.998	0.999	0.999	5.04
Both		k = 2, metric = manhattan, weights = distance	0.965	0.966	0.965	0.965	0.965	0.983	0.31
LV	Single Layer Neural Network	units = 50, learning rate = 0.1	0.992	0.993	0.992	0.992	0.992	0.996	0.07
OB		units = 10, learning rate = 0.1	0.992	0.991	0.994	0.992	0.994	0.996	0.07
Both		units = 200, lr = 0.01	0.954	0.944	0.951	0.947	0.967	0.977	0.06

* Speed refers to the amount of $10^{5}$ predictions/second the algorithm can perform.

**Table 11:** Volunteers task scores.

Sub-Task	Completion of Sub-Tasks	Speed of Completion	Movement Quality	Skillfulness of Prosthesis Use	Independence
**Volunteer Code**	**OB | LV**	**OB | LV**	**OB | LV**	**OB | LV**	**OB | LV**
Put on shirt	2 | 1	2 | 1	2 | 1	0 | 1	2 | 2
Take off shirt	2 | 1	2 | 1	2 | 1	1 | 1	3 | 2
Buttoning buttons	3 | 2	2 | 2	2 | 2	0 | 0	2 | 2
**Volunteer Code**	**OB | LV**	**OB | LV**	**OB | LV**	**OB | LV**	**OB | LV**
Running zipper	3 | 0	2 | 0	2 | 0	2 | 0	1 | 0
Tie shoelace	3 | 2	2 | 2	1 | 1	2 | 2	2 | 2
Drink water	3 | 0	3 | 0	2 | 0	2 | 2	2 | 1
Use cutlery	1 | 0	1 | 1	2 | 1	1 | 1	1 | 1
Pour liquid	2 | 2	2 | 2	2 | 2	1 | 2	2 | 2
Write initials	3 | 2	3 | 3	2 | 1	2 | 1	1 | 1
Dial a number	2 | 0	2 | 2	3 | 2	2 | 1	1 | 2
Fold clothes	2 | 2	2 | 3	3 | 2	1 | 2	2 | 2
Grab on shelf	1 | 0	1 | 0	1 | 0	1 | 0	1 | 0

**Table 12:** Comparative analysis of sEMG classification algorithms.

Aspect	Libra Neurolimb	Hassan et al. [50]	Phukpattaranont et al. [55]	Shen et al. [54]	Chen et al. [56]	Said et al. [57]
SFR *	100 Hz	200 Hz	1024 Hz	10 KHz	200 Hz	200 Hz
Gestures	3	7	14	41	5	4
Channels	2	8	6	8	8	8
Accuracy (%)	SVM 99.2,	SVM 95.26	SVM 93, LC 94, NB 90,			
	k-NN 99.8	LDA 92.58	KNN 93, RBF-ELM 93,	74	89	89.93
		K-NN 86.41	AW-ELM, NN99			
Data transfer	real-time	online mode **	offline	real-time	offline	real-time
Signal typye	sEMG	sEMG	sEMG	sEMG	sEMG	sEMG

* SFR: Superficial Frequency Rate. ** It possesses offline mode too.

## Data Availability

Due to privacy restrictions with the participating volunteers, the datasets generated for the model during the research are kept confidential.

## Abbreviations

The following abbreviations are used in this manuscript:

AM-ULA	Activities Measure for Upper Limb Amputees
BCI	Brain–Computer Interface
DOF	Degree(s) of Freedom
EEG	Electroencephalography
EMG	Electromyography
FDM	Fused Deposition Modeling
IoT	Internet of Things
OSC	Open Sound Control
PLA	Polylactic acid
sEMG	Surface Electromyography
SVM	Support Vector Machine
TRL	Technology Readiness Level
UDP	User Datagram Protocol
